# Emerging genetic mechanisms of glomerular proteinuria: a podocyte-centered multi-layer network perspective

**DOI:** 10.3389/fgene.2026.1934937

**Published:** 2026-09-21

**Authors:** Meijia Fu, Jiayi Lin, Cong Wang, Xiaoyuan Wang

**Affiliations:** 1 Shandong First Medical University (Shandong Academy of Medical Sciences), Jinan, Shandong, China; 2 Shandong Provincial Hospital Affiliated to Shandong First Medical University, Jinan, Shandong, China

**Keywords:** cytoskeletal regulation, genetic mechanisms, glomerular proteinuria, membrane trafficking, mitochondrial homeostasis, nuclear pore complex, podocyte homeostasis, podocyte injury

## Abstract

Podocyte injury is widely recognized as a central driver of glomerular proteinuria and progression to chronic kidney disease. Although proteinuria can originate from multiple components of the glomerular filtration barrier, genetic and experimental studies increasingly implicate podocyte dysfunction as a dominant and convergent mechanism in many disease contexts. In recent years, the application of next-generation sequencing has broadened the spectrum of genes linked to proteinuric kidney disorders. These genes are no longer confined to structural elements of the slit diaphragm or cytoskeleton but also involve pathways governing vesicular trafficking, nuclear regulation, mitochondrial function, and interactions with the extracellular matrix. Despite their functional diversity, alterations in these pathways often produce overlapping clinical phenotypes, suggesting convergence at the level of cellular homeostasis rather than at a single molecular endpoint. Experimental observations further indicate that podocytes maintain their structural and functional integrity through coordinated regulation across multiple cellular compartments. Mechanical cues originating from the glomerular basement membrane are interpreted through cytoskeletal signaling; membrane trafficking processes determine the localization and turnover of key slit diaphragm proteins; nuclear mechanisms shape transcriptional responses; mitochondrial systems contribute to metabolic adaptation under stress conditions. Perturbations in any of these processes may not remain localized but can extend across interconnected pathways, gradually impairing filtration barrier function. Current evidence therefore favors a multi-layer network perspective in which glomerular proteinuria reflects a breakdown of coordinated podocyte homeostasis across interconnected cellular compartments.

## 1 Introduction

Podocytopathies represent a major cause of glomerular proteinuria and are strongly associated with progression to chronic kidney disease. Genetic and experimental studies have consistently identified podocyte dysfunction as a central determinant of disease pathogenesis.

Early genetic discoveries established a structural framework in which mutations affecting slit diaphragm proteins and cytoskeletal regulators, including *NPHS1* (OMIM: 602716), *NPHS2* (OMIM: 604766), *ACTN4* (OMIM: 604638), and *INF2* (OMIM: 610982), compromise filtration barrier integrity and lead to proteinuria (Kaplan et al., 2000; Brown et al., 2010; Boute et al., 2000; Kestilä et al., 1998). In parallel, variants in genes encoding glomerular basement membrane components, including *COL4A3* (OMIM: 120070), *COL4A4* (OMIM: 120131), *COL4A5* (OMIM: 303630) and *LAMB2* (OMIM: 150325), have been identified in patients with hereditary kidney disease and are associated with structural and functional alterations of the filtration barrier (Zhu HT. et al., 2019; Qian et al., 2022; Zhang et al., 2023; Daga et al., 2025).

Large-scale sequencing studies have further highlighted the substantial genetic heterogeneity of proteinuric kidney disease. Whole-exome sequencing of a multicenter pediatric nephrotic syndrome cohort identified monogenic causes in approximately 30% of patients with idiopathic steroid-resistant nephrotic syndrome (Jiao et al., 2022), while a whole-genome sequencing study achieved a molecular diagnostic yield of 56.1% in families with steroid-resistant nephrotic syndrome and identified both established and previously unreported disease-associated variants (Soliman et al., 2025). These findings illustrate the increasing value of genome-scale approaches for defining the molecular basis of podocytopathies.

Complementing disease-variant discovery, recent single-cell and spatial transcriptomic studies have provided a broader developmental context for interpreting genetic perturbations. Levinsohn et al. generated a spatially resolved atlas of human kidney development and identified cell-fate plasticity and spatially organized signaling associated with nephron lineage specification (Levinsohn et al., 2026). These findings provide a systems-level framework for understanding how developmental programs and the local microenvironment contribute to the establishment of podocyte identity.

Together, these genetic and functional-genomic advances have expanded the conceptual landscape of podocytopathy beyond classical structural components. Podocytopathy-associated genes can be broadly classified into several functional categories, including slit diaphragm and filtration barrier organization, actin cytoskeleton and structural integrity, podocyte transcriptional and developmental regulation, cell adhesion and extracellular matrix interactions, intracellular trafficking, and mitochondrial homeostasis. Although these genes participate in diverse biological processes, their dysfunction may converge on impaired podocyte homeostasis and disruption of the glomerular filtration barrier. These interconnected functional layers and their convergence on podocyte homeostasis are summarized in Figure 1.

**FIGURE 1:**
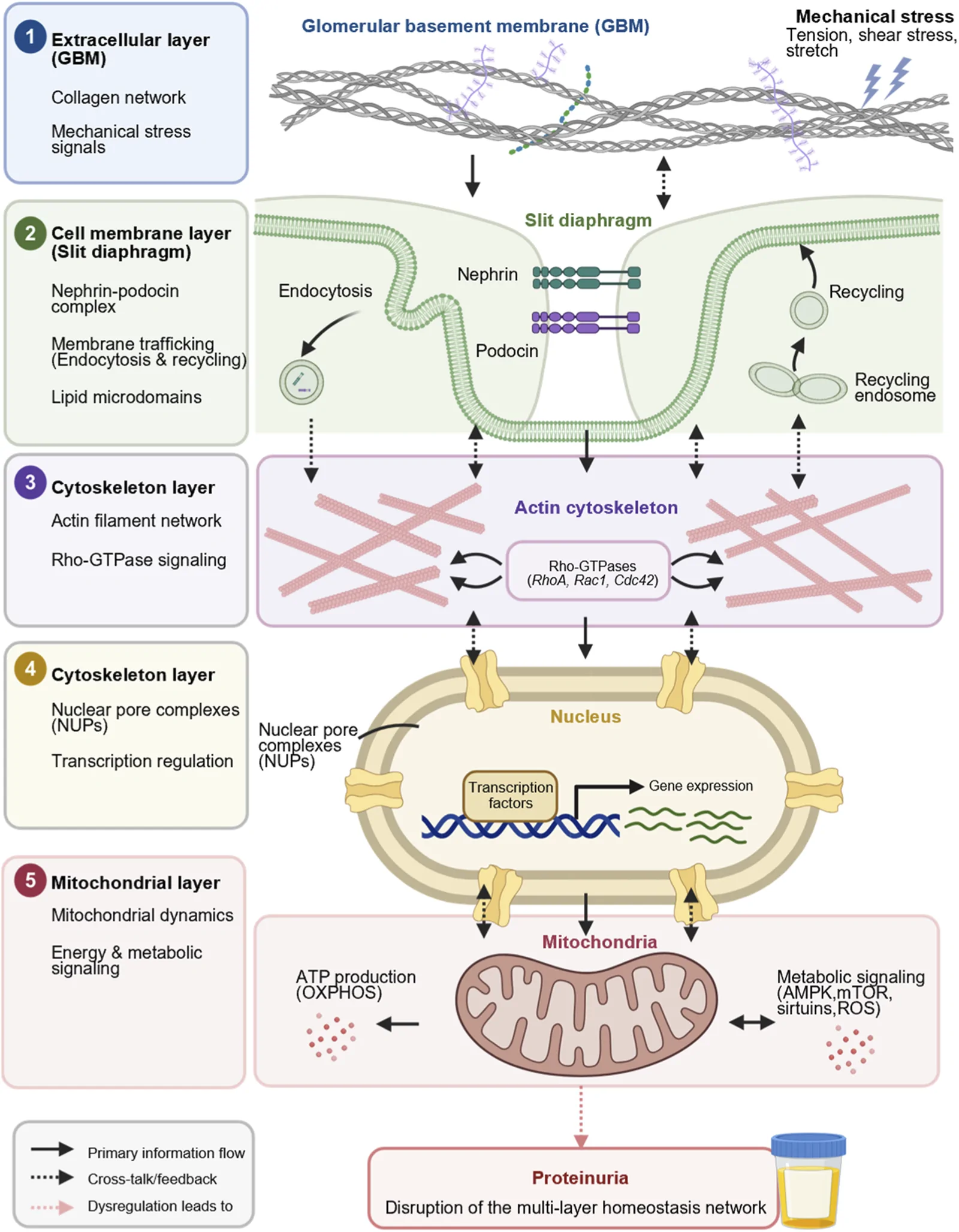
Multi-layer organization of podocyte homeostasis and its disruption in glomerular proteinuria. The glomerular filtration barrier is maintained by coordinated regulation across multiple cellular and extracellular layers. The extracellular layer consists of the glomerular basement membrane (GBM), which provides structural support and transduces mechanical stress signals. The cell membrane layer is defined by the slit diaphragm, where the nephrin–podocin complex undergoes dynamic turnover through endocytosis and recycling pathways. Beneath this, the actin cytoskeleton maintains foot process architecture through Rho-GTPase–dependent signaling. Nuclear regulation integrates transcriptional and stress-response programs via nuclear pore complexes and transcription factors. Mitochondrial function supports cellular energy production and metabolic signaling, including reactive oxygen species (ROS) generation and AMPK/mTOR pathways. Crosstalk between these layers contributes to the maintenance of podocyte integrity, whereas disruption of this multi-layered homeostatic network ultimately leads to proteinuria.

Emerging genetic evidence has expanded the spectrum of implicated genes to include regulators of cytoskeletal signaling such as *NOS1AP* (Nitric oxide synthase 1 adaptor protein, OMIM: 605551) and *DAAM2* (OMIM: 606627) (Majmundar et al., 2021; Buerger et al., 2025; Schneider et al., 2020), Rho-GTPase–associated proteins including *MYO9A* (OMIM: 604875) and additional regulators such as *ARHGAP32* (OMIM: 608541) (Li et al., 2021; Chen et al., 2025), vesicular trafficking components such as *TBC1D8B* (OMIM: 617138) (Kampf et al., 2019; Milosavljevic et al., 2022), nuclear pore–associated proteins including *TRIM8* (OMIM: 616093) and NUP family members (Weng et al., 2021; Rogg et al., 2022; Han et al., 2024; Li Y. et al., 2024; Liu et al., 2025).

Furthermore, transcriptional regulators such as *WT1* (OMIM: 607102) and *LMX1B* (OMIM: 602575) play essential roles in podocyte differentiation and maintenance of mature filtration barrier function, highlighting the contribution of gene regulatory networks to podocyte disease. Mitochondrial regulators such as *MTX2* (OMIM: 602614) (Li T. et al., 2024) further expand the mechanistic landscape. In addition, cilia-associated *TTC21B* (OMIM: 613819) and kidney-risk *APOL1* (OMIM: 603743) variants associated with FSGS/ESKD illustrate additional genetic mechanisms contributing to podocyte dysfunction (Bezdíčka et al., 2021; Genovese et al., 2010; Tzur et al., 2010).

Accumulating evidence further indicates that developmental regulation itself can contribute to podocytopathy. Pathogenic *SMAD2* variants have been shown to impair podocyte differentiation and glomerular filtration barrier formation (Bhattacharya et al., 2025), extending the genetic framework from mechanisms that maintain mature podocytes to developmental programs required for establishing functional podocyte identity.

Although this review primarily focuses on emerging genetic mechanisms underlying glomerular proteinuria, podocyte dysfunction cannot be fully understood through genetic alterations alone. Increasing evidence indicates that acquired insults, particularly metabolic stress associated with kidney disease, may influence podocyte function through molecular pathways that overlap with those affected by genetic variants (Dai et al., 2017; Alicic et al., 2017). These acquired stressors can perturb key aspects of podocyte homeostasis, including filtration barrier integrity and cytoskeletal organization, thereby contributing to podocyte injury and proteinuria (Alicic et al., 2017; Faul et al., 2007). Therefore, non-genetic mechanisms are discussed in this review not as independent disease categories, but as complementary perturbations that help define the convergent molecular architecture underlying podocyte injury.

A comprehensive functional overview of representative podocytopathy-associated genes, their biological roles, and their contributions to glomerular filtration barrier dysfunction is provided in Table 1.

**TABLE 1:** Functional classification and biological roles of podocytopathy-associated genes involved in glomerular filtration barrier dysfunction.

Gene	Biological function	Contribution to podocyte dysfunction	References
Actin cytoskeleton and structural integrity			
***ACTN4***	Encodes α-actinin-4, an actin-binding protein that maintains mechanical stability of the podocyte cytoskeleton	Disease-associated variants alter actin-binding properties and weaken cytoskeletal stability, resulting in foot process abnormalities and familial FSGS	Kaplan et al., 2000
***INF2***	Encodes a formin protein regulating actin polymerization and depolymerization dynamics	Mutations disturb actin remodeling processes and impair podocyte structural adaptation	Brown et al., 2010
***NOS1AP***	Regulates CDC42/DIAPH-dependent actin remodeling pathways in podocytes	Loss of NOS1AP function disrupts actin organization and causes foot process abnormalities and proteinuria	Majmundar et al., 2021
***DAAM2***	Formin-related protein involved in wnt signaling and actin assembly	Biallelic variants have been associated with steroid-resistant nephrotic syndrome, affecting actin organization and possibly podocyte development	Schneider et al., 2020
***KANK2***	Scaffold protein localized at focal adhesions that connects integrin-mediated adhesion complexes with the actin cytoskeleton	Mutations disrupt cytoskeletal–adhesion coupling and impair podocyte attachment to the glomerular basement membrane	Gee et al., 2015; Xu et al., 2021
***MYO9A***	Unconventional myosin containing Rho-GTPase activating activity and involved in cytoskeletal regulation	Rare variants have been associated with altered podocyte migration and stress fiber organization, although pathogenic evidence remains limited	Li et al., 2021
***ARHGAP32***	Regulator of CDC42 signaling involved in rho-GTPase-mediated cytoskeletal control	Pathogenic variants cause abnormal rho signaling, reduced nephrin/F-actin expression, and podocyte injury	Chen et al., 2025
Slit diaphragm, filtration barrier, and membrane protein homeostasis			
***NPHS1***	Encodes nephrin, a core slit diaphragm protein forming the filtration barrier complex	Variants impair nephrin biosynthesis, maturation, trafficking, and slit diaphragm assembly, leading to severe proteinuria	Kestilä et al., 1998; Li et al., 2001; Ohmori et al., 2021
***NPHS2***	Encodes podocin, a key component of the nephrin–podocin slit diaphragm complex	Disease-associated variants affect podocin stability, intracellular trafficking, and membrane localization	Boute et al., 2000; Roselli et al., 2010; Dorison et al., 2023
***KIRREL1 (NEPH1)***	Encodes a cell adhesion molecule participating in slit diaphragm assembly and stabilization	Disruption affects slit diaphragm organization and filtration barrier integrity	Donoviel et al., 2001
***CD2AP***	Scaffold protein linking slit diaphragm proteins with intracellular signaling pathways and actin cytoskeleton	Loss or pathogenic variants disrupt slit diaphragm–cytoskeleton organization and cause nephrotic syndrome/FSGS	Shih et al., 1999; Shih et al., 2001; Kim et al., 2003; Takano et al., 2019
***MAGI2***	Multi-domain scaffold protein involved in slit diaphragm molecular organization	Loss or pathogenic variants disrupt SD organization and Rap1-dependent cytoskeletal signaling	Zhang et al., 2021; Bierzynska et al., 2017; Shirata et al., 2017; Zhu et al., 2019a
***TRPC6***	Calcium-permeable channel involved in slit diaphragm-associated signaling	Pathogenic variants cause abnormal cation signaling, with delayed channel inactivation contributing to FSGS	Reiser et al., 2005; Winn et al., 2005; Krall et al., 2010; Okada et al., 2025
***TBC1D8B***	Rab-related regulator involved in endosomal maturation, nephrin trafficking, and recycling	Loss of function disrupts nephrin endocytosis and recycling, reducing functional slit diaphragm protein availability	Kampf et al., 2019; Milosavljevic et al., 2022
***AP2B1***	Component of the AP-2 adaptor complex mediating clathrin-dependent endocytosis	Impairs membrane protein internalization and trafficking regulation	Kadlecova et al., 2017
***PICALM***	Endocytic adaptor involved in clathrin assembly and vesicle formation	Disturbs intracellular trafficking processes required for membrane protein turnover	Tebar et al., 1999
***OCRL***	Regulates phosphoinositide metabolism and endosomal dynamics	Alters endosomal regulation and intracellular transport pathways	Vicinanza et al., 2011
***EHD1***	Regulator of recycling endosome formation and membrane protein recycling	Impairs recycling of membrane proteins and contributes to slit diaphragm instability	Lee et al., 2015
***EXOC4***	Component of the exocyst complex involved in polarized vesicle delivery	Disrupts nephrin and NEPH1 localization to the plasma membrane	Nihalani et al., 2019
***EXOC5***	Exocyst complex component involved in vesicle docking and membrane targeting	Affects slit diaphragm protein distribution and podocyte foot process maintenance	Nihalani et al., 2019
Podocyte transcriptional, developmental, and nuclear regulation			
***WT1***	Transcription factor essential for podocyte differentiation and maintenance of mature podocyte identity	Mutations disrupt podocyte developmental programs and compromise filtration barrier function	Dong et al., 2015
***LMX1B***	Transcriptional regulator involved in podocyte differentiation and maturation	Alters expression of genes required for podocyte development and filtration barrier maintenance	Burghardt et al., 2013
***SMAD2***	Regulates TGF-β signaling during mesodermal development and podocyte differentiation	Mutations disrupt podocyte differentiation programs and impair foot process development and filtration barrier formation	Bhattacharya et al., 2025
***TRIM8***	Nuclear regulatory protein involved in nuclear organization and transcriptional responses	Variants disrupt nuclear localization and transcriptional regulation associated with podocyte dysfunction	Weng et al., 2021
***NUP133***	Nuclear pore complex component involved in nuclear pore assembly and transcriptional regulation	Deficiency alters nuclear pore integrity, transcriptional profiles, and cytoskeletal organization	Rogg et al., 2022
***NUP93***	Nuclear pore protein involved in nuclear transport	Biallelic variants are associated with early-onset steroid-resistant nephrotic syndrome	Braun et al., 2016; Han et al., 2024
***NUP107***	Nuclear pore component involved in nuclear pore complex organization	Biallelic variants are associated with SRNS/FSGS and progression to ESKD	Han et al., 2024
***NUP160***	Nuclear pore protein involved in podocyte homeostasis	Loss of function reduces CDC42 activity and affects cytoskeletal regulation and filtration barrier maintenance	Han et al., 2024; Li et al., 2024a; Liu et al., 2025
Mitochondrial homeostasis and inter-organelle regulation			
***MTX2***	Mitochondrial membrane protein involved in mitochondrial protein import and cristae organization	Deficiency disrupts mitochondrial structure, adhesion, migration, and podocyte function	Li et al., 2024b
***APOL1***	Membrane-associated protein with monovalent cation transport activity involved in cellular and organelle homeostasis	G1/G2 risk variants promote podocyte injury through abnormal cation transport and organelle/metabolic dysfunction	Genovese et al., 2010; Beckerman et al., 2017; Ma et al., 2017; Ge et al., 2021; Datta et al., 2024
***COQ8B***	Protein involved in coenzyme Q biosynthesis and mitochondrial metabolism	Impairs mitochondrial energy metabolism and contributes to steroid-resistant nephrotic syndrome	Zhang et al., 2024
***PDSS1***	Enzyme involved in coenzyme Q synthesis	Alters mitochondrial respiratory function and cellular energy balance	Habib et al., 2025
***NDUFS4***	Regulator of mitochondrial respiratory chain complex I organization	Experimental evidence suggests a role in mitochondrial organization and oxidative metabolism, mainly from acquired kidney disease models	Mise et al., 2024
***MFN2***	Regulator of mitochondria–ER contact sites and mitochondrial dynamics	Downregulation disrupts ER–mitochondria communication and promotes podocyte injury in metabolic disease models	Cao et al., 2021
Cell adhesion, extracellular matrix, and GBM–podocyte interactions			
***COL4A3***	Encodes collagen IV α3 chain, a major structural component of the glomerular basement membrane	Variants alter GBM composition and filtration barrier structure, causing hereditary proteinuria	Zhang et al., 2023; Daga et al., 2025; Puapatanakul et al., 2024
***COL4A4***	Encodes collagen IV α4 chain within the GBM collagen network	Disrupts GBM organization and contributes to alport syndrome-related disease	Zhang et al., 2023; Puapatanakul et al., 2024
***COL4A5***	Encodes collagen IV α5 chain forming the collagen IV α3α4α5 network	Mutations impair GBM integrity and cause alport syndrome	Qian et al., 2022; Zhang et al., 2023; Puapatanakul et al., 2024
***LAMB2***	Encodes laminin β2, a component of GBM laminin complexes	Alters GBM organization and podocyte attachment	Zhu et al., 2019b
***EPB41L5***	Regulates extracellular matrix assembly and adhesion complex organization	Loss of function impairs podocyte adhesion and structural stability	Maier et al., 2021
***CD151***	Tetraspanin involved in integrin-mediated adhesion complexes	Disrupts podocyte–GBM attachment and mechanical stability	Naylor et al., 2022
***FAT1***	Regulator of cell polarity and Hippo signaling pathways	Mutations alter adhesion regulation and mechanosensory signaling	Fabretti et al., 2021
***CRB2***	Polarity-associated protein involved in mechanosensitive signaling	Mutations affect YAP-dependent responses and podocyte adaptation to mechanical stress	Sun et al., 2025

All gene names are shown in bold for consistency and to improve readability.

In this review, we define classical podocytopathy-associated genes as genes with well-established pathogenic mechanisms supported by extensive clinical evidence and multiple reported cases. In contrast, emerging genes refer to newly implicated genes or newly recognized disease mechanisms for which genetic and/or functional evidence suggests a contribution to glomerular proteinuria, but clinical validation remains limited. Within this framework, this review examines how genetically diverse perturbations affecting distinct functional layers of podocyte biology may converge on shared mechanisms of podocyte dysfunction and glomerular proteinuria.

## 2 Dysregulation of cytoskeletal homeostasis and emerging genes

### 2.1 Classical mechanisms of cytoskeletal regulation

The foot processes of podocytes form the structural basis of the glomerular filtration barrier and rely on a highly organized yet continuously remodeled actin cytoskeleton. Genetic studies have established that disruption of cytoskeletal integrity can directly impair foot process architecture, leading to filtration barrier failure and proteinuria. Mutations in *ACTN4* alter the interaction between α-actinin-4 and F-actin, thereby weakening mechanical stability and giving rise to autosomal dominant familial FSGS (1). Similarly, *INF2*, a member of the formin family, regulates actin polymerization dynamics; pathogenic variants disturb the balance between actin assembly and disassembly, further implicating cytoskeletal remodeling in disease pathogenesis (Brown et al., 2010).

Additional evidence highlights the importance of Rho-GTPase signaling in maintaining podocyte structure. *ARHGAP24* (OMIM: 610586) modulates RAC1 activity, and its dysfunction has been associated with altered cytoskeletal organization and proteinuria phenotypes (Akilesh et al., 2011). Collectively, these findings established a mechanistic link between cytoskeletal disruption, foot process effacement, and proteinuria, although the cytoskeleton was largely viewed as a structural scaffold within this framework.

### 2.2 Emerging genes identified in recent studies

#### 2.2.1 Emerging disease genes with functional validation

Recent work combining genetic analysis with experimental validation has identified several genes with stronger evidence for involvement in podocyte cytoskeletal regulation. Among these, *NOS1AP* has attracted particular attention. Recessive variants identified through exome sequencing in early-onset nephrotic syndrome families have been shown to reproduce proteinuria and foot process abnormalities in both murine models and human kidney organoids. Mechanistic studies suggest that loss of *NOS1AP* function impairs CDC42 and DIAPH-dependent actin remodeling, thereby affecting structural stability of podocyte processes (Majmundar et al., 2021).

Further complexity has emerged from the identification of a kidney-enriched splice isoform located within the *NOS1AP-C1orf226* intergenic region. Variants affecting this transcript have been associated with podocytopathy and can influence transcript processing (Buerger et al., 2025). Functional studies indicate that disruption of this isoform leads to proteinuria and may retain partial responsiveness to RAAS inhibition (Buerger et al., 2025). At present, evidence remains limited to a small number of cases and experimental systems, and the contribution of such variants at the population level requires further clarification. Nonetheless, these findings point to a potential role for noncoding variation and splicing regulation in cytoskeletal control.

#### 2.2.2 Emerging genes with moderate supporting evidence

*DAAM2* represents another gene with emerging but still developing evidence. It encodes a formin-related protein involved in Wnt signaling and actin assembly. Biallelic variants have been identified in families with steroid-resistant nephrotic syndrome, and functional assays suggest an effect on actin organization. In *Xenopus* models, loss of *DAAM2* leads to glomerular developmental abnormalities, whereas expression of wild-type *DAAM2* mRNA partially restores the phenotype (Schneider et al., 2020).

The phenotypic spectrum of *DAAM2*-associated disease has been further extended by a recent report of two siblings carrying a homozygous nonsense variant (p.Arg66Ter), who presented with steroid-resistant nephrotic syndrome accompanied by short stature and dysmorphic features, raising the possibility of extrarenal involvement in some patients (Pragna et al., 2024).

Preliminary studies in *Drosophila* nephrocytes suggest that *DAAM2* deficiency may influence nephrin endocytosis and basal autophagy, raising the possibility that *DAAM2* may affect slit diaphragm stability not only through actin assembly but also through membrane protein turnover, although this mechanism requires validation in mammalian podocyte systems (Lang et al., 2025).

*KANK2* (OMIM: 614273) provides an additional example of cytoskeleton-associated regulation at the cell–matrix interface. The mutation of the *KANK2* gene was first reported to cause nephrotic syndrome in 2015 (35). *KANK2* encodes a scaffold protein localized to focal adhesions, where it contributes to the coupling between integrin-mediated adhesion complexes and the actin cytoskeleton. Pathogenic variants identified in patients with nephrotic syndrome have been shown to disrupt the interaction between *KANK2* and *KIF21A* through abnormal competitive binding, thereby impairing cytoskeletal–adhesion coupling and destabilizing podocyte attachment.

Beyond this specific interaction, *KANK2* is thought to participate in organizing adhesion-associated signaling platforms that regulate force transmission across the GBM–podocyte interface. Disruption of *KANK2* function may therefore alter mechanosensory signaling and downstream cytoskeletal dynamics (Gee et al., 2015; Xu et al., 2021; Lennon et al., 2014). Within a multi-layer network framework, *KANK2* can be positioned at the interface between extracellular matrix sensing and intracellular structural regulation, linking mechanical inputs to cytoskeletal responses (Xu et al., 2021).

#### 2.2.3 Candidate genes with limited evidence

*MYO9A* encodes an unconventional myosin with Rho-GTPase activating protein activity and has been proposed as a candidate regulator of cytoskeletal dynamics. Rare variants identified in small cohorts of FSGS patients have been associated with altered podocyte migration and stress fiber organization (Li et al., 2021). However, current evidence is largely restricted to limited case series and *in vitro* studies, and its role as a disease-causing gene has not yet been firmly established.

More recently, a heterozygous *ARHGAP32* missense variant (p.L754V) has been implicated in autosomal dominant FSGS. Functional analyses indicate that the mutant protein exhibits increased CDC42-binding affinity, resulting in Rho-GTPase dysregulation, reduced nephrin and F-actin expression in podocytes, and proteinuria with glomerular injury in a CRISPR/Cas9 knock-in mouse model (Chen et al., 2025). These findings further support the concept that aberrant activation of Rho-GTPase signaling, rather than simple loss-of-function mechanisms alone, may destabilize podocyte cytoskeletal homeostasis.

#### 2.2.4 Membrane trafficking and endocytic regulation as emerging mechanisms of cytoskeletal homeostasis

In addition to newly identified genes, further mechanistic insights have expanded the functional roles of previously implicated genes. *TBC1D8B*, initially associated with nephrin trafficking, has been shown to regulate endosomal maturation, nephrin turnover, and Rab5-dependent endocytosis (Kampf et al., 2019; Milosavljevic et al., 2022). Because nephrin-dependent actin reorganization in podocytes is mediated by the recruitment of Nck adaptor proteins to phosphorylated motifs within the nephrin cytoplasmic tail (Jones et al., 2006), *TBC1D8B* alterations may influence podocyte cytoskeletal integrity through disruption of membrane protein homeostasis. Thus, impaired nephrin trafficking may indirectly affect actin organization and foot process maintenance by altering the availability of functional slit diaphragm signaling complexes (Kampf et al., 2019; Milosavljevic et al., 2022; Jones et al., 2006).

Beyond *TBC1D8B*, several genes involved in endocytosis and vesicular trafficking have established roles in endocytosis and vesicular trafficking and are therefore considered potential regulators of podocyte homeostasis. *AP2B1*, encoding the β2-adaptin subunit of the AP-2 adaptor complex, contributes to clathrin-mediated endocytosis through adaptor complex assembly and cargo recruitment, whereas *PICALM* functions as an endocytic adaptor involved in clathrin assembly and endocytic vesicle formation (Tebar et al., 1999; Kadlecova et al., 2017). In addition, *OCRL* regulates phosphoinositide metabolism and endosomal dynamics, whereas *EHD1* contributes to recycling endosome formation and membrane protein recycling (Lee et al., 2015; Vicinanza et al., 2011). Although direct genetic evidence linking these genes to human podocytopathy remains limited, their involvement in membrane trafficking pathways highlights the importance of intracellular transport and membrane protein turnover in maintaining slit diaphragm stability and podocyte function.

Together, these findings indicate that membrane trafficking is not an independent cellular process but an integrated component of cytoskeletal regulation. Consistent with this concept, cytoskeletal regulators such as *INF2* further illustrate the reciprocal relationship between actin dynamics and membrane-associated processes, as alterations in cytoskeletal remodeling can impair podocyte structural adaptation (Brown et al., 2010).

Although increasing numbers of podocytopathy-associated genes have been identified, the pathogenic relevance of individual variants requires integration of clinical phenotype, functional validation, and genetic evidence. Representative disease-associated variants of emerging podocyte genes with available experimental support are summarized in Table 2.

**TABLE 2:** Representative podocytopathy-associated variants and their experimental evidence supporting pathogenic mechanisms.

Gene	Variant	Protein change	Associated phenotype	Functional validation (experimental evidence)	Evidence level (ACMG classification)	References
*KANK2*	c.541A>G	p.Ser181Gly	Childhood-onset SSNS; minimal change disease	Yes (cultured podocytes, *Drosophila* nephrocytes, zebrafish)	LP(PS3+PM2+PP3+PP4)	Gee et al., 2015
*KANK2*	c.2051C>T	p.Ser684Phe	Childhood-onset SSNS	Yes (cultured podocytes, *Drosophila* nephrocytes, zebrafish)	LP(PS3+PM2+PP3+PP4)	Gee et al., 2015
*MYO9A*	c.2101C>T	p.Arg701Ter	Familial FSGS; proteinuria; progressive renal dysfunction	Yes (knock-in mice, cultured podocytes)	LP(PS3+PM2+PP3+PP4)	Li et al., 2021
*NOS1AP*	c.345 + 3T>G	Splice variant	Early-onset steroid-resistant nephrotic syndrome	Yes (cultured podocytes, kidney organoids, Nos1ap mutant mice)	LP(PS3+PM2+PP3+PP4)	Majmundar et al., 2021
*NOS1AP*	c.428G>A	p.Cys143Tyr	Early-onset steroid-resistant nephrotic syndrome	Yes (kidney organoids, mouse model)	LP(PS3+PM2+PP3+PP4)	Majmundar et al., 2021
*NOS1AP*	c.271_329del	p.Val91_110delfs*7	Albuminuria; podocytopathy	Yes (Nos1ap mutant mice)	P(PVS1+PS3)	Buerger et al., 2025
*DAAM2*	c.361C>T	p.Gln121*	Steroid-resistant nephrotic syndrome (SRNS)	Yes (cultured podocytes, actin assembly assay, *Xenopus* CRISPR knockout model)	P(PVS1+PS4)	Schneider et al., 2020
*DAAM2*	c.1004G>A	p.Arg335His	Steroid-resistant nephrotic syndrome (SRNS)	Yes (cultured podocytes, rescue assay, actin remodeling analysis)	LP(PS3+PM2+PP3+PP4)	Schneider et al., 2020
*DAAM2*	c.1333T>C	p.Cys445Arg	Steroid-resistant nephrotic syndrome (SRNS)	Yes (podocyte migration and filopodia rescue assay)	LP(PS3+PM2+PP3+PP4)	Schneider et al., 2020
*DAAM2*	c.1745C>T	p.Pro582His	Steroid-resistant nephrotic syndrome (SRNS)	Yes (cultured podocytes, actin assembly assay)	LP(PS3+PM2+PP3+PP4)	Schneider et al., 2020
*DAAM2*	c.308C>T	p.Ser103Leu	Steroid-resistant nephrotic syndrome (SRNS)	Yes (functional rescue and actin regulation assays)	LP(PS3+PM2+PP3+PP4)	Schneider et al., 2020
*ARHGAP32*	p.Leu754Val (L754V)	Leu754Val	Focal segmental glomerulosclerosis (FSGS); proteinuria; renal dysfunction	Yes (cultured podocytes, COS-7 cells, CRISPR/Cas9 knock-in mice)	LP(PS3+PM2+PP3+PP4)	Cheng et al., 2024
*TBC1D8B*	c.2338A>T	p.Thr780Ser	Nephrotic syndrome/SRNS	Yes (podocyte-like *Drosophila* nephrocytes, RAB11 and nephrin interaction assays)	LP(PS3+PM2+PP3+PP4)	Kampf et al., 2019; Milosavljevic et al., 2022
*TBC1D8B*	c.190C>T	p.Arg64Cys	Nephrotic syndrome/SRNS	Yes (functional analysis of RAB11/nephrin binding)	LP(PS3+PM2+PP3+PP4)	Kampf et al., 2019; Milosavljevic et al., 2022
*TBC1D8B*	c.1316T>G	p.Phe439Cys	Nephrotic syndrome/SRNS	Yes (patient-derived mutant analysis, *Drosophila* model)	LP(PS3+PM2+PP3+PP4)	Kampf et al., 2019; Milosavljevic et al., 2022
*TBC1D8B*	c.1383G>A	p.Trp461*	Nephrotic syndrome/SRNS	Yes (functional analysis in podocyte-related models)	LP(PS3+PM2+PP3+PP4)	Kampf et al., 2019; Milosavljevic et al., 2022
*TBC1D8B*	c.1030C>T	p.Arg344*	Nephrotic syndrome/SRNS	Yes (functional analysis in *Drosophila* nephrocytes)	LP(PS3+PM2+PP3+PP4)	Kampf et al., 2019; Milosavljevic et al., 2022
*TBC1D8B*	c.2089delA	p.Asp698Metfs*6	Adult-onset FSGS, nephrotic syndrome	No direct functional assay; genetic + clinical evidence	P(PVS1+PM2+PP4)	Hu et al., 2024
*TRIM8*	c.1375C>T	p.Gln459*	SRNS/FSGS + developmental delay + epilepsy	Yes (protein localization, podocyte/neuronal cells)	P(PVS1+PS3+PM2+PP4)	Weng et al., 2021
*TRIM8*	c.1163delT	p.Phe388Serfs*2	SRNS/FSGS + developmental delay + epilepsy	Yes (protein localization, podocyte/neuronal cells)	P(PVS1+PS3+PM2+PP4)	Weng et al., 2021
*TRIM8*	c.1231C>T	p.Gln411*	SRNS/FSGS + developmental delay + epilepsy	Yes (protein localization, podocyte/neuronal cells)	P(PVS1+PS3+PM2+PP4)	Weng et al., 2021
*TRIM8*	c.1240C>T	p.Gln414*	SRNS/FSGS + developmental delay + epilepsy	Yes (protein localization, podocyte/neuronal cells)	P(PVS1+PS3+PM2+PP4)	Weng et al., 2021
*TRIM8*	c.1333C>T	p.Gln445*	SRNS/FSGS + developmental delay + epilepsy	Yes (protein localization, podocyte/neuronal cells)	P(PVS1+PS3+PM2+PP4)	Weng et al., 2021
*NUP107*	c.1199G>A	p.Gly400Glu	Steroid-resistant nephrotic syndrome (SRNS); FSGS; progression to ESKD	Yes (clinical genetic analysis; previous zebrafish/podocyte functional studies of NUP107 deficiency)	LP(PS3+PM2+PP3+PP4)	Han et al., 2024
*NUP107*	c.580C>T	p.Arg194*	SRNS; FSGS; progression to ESKD	Yes (clinical genetic analysis; functional evidence from NUP107 deficiency models)	P(PVS1+PS3+PM2+PP4)	Han et al., 2024
*NUP107*	c.2564delC	p.Pro855fsTer*23	SRNS; FSGS; progression to ESKD	Yes (clinical genetic analysis; functional evidence from NUP107 deficiency models)	P(PVS1+PS3+PM2+PP4)	Han et al., 2024
*NUP107*	c.2753C>T	p.Pro918Leu	SRNS; FSGS; progression to ESKD	Yes (clinical genetic analysis; functional evidence from NUP107 deficiency models)	LP(PS3+PM2+PP3+PP4)	Han et al., 2024
*NUP160*	c.3656T>G	p.Leu1219Trp	Early-onset steroid-resistant nephrotic syndrome (SRNS); FSGS; CKD progression	Yes (podocyte-specific Nup160 knockout mice; primary podocyte studies)	LP(PS3+PM2+PP3+PP4)	Han et al., 2024; Li et al., 2024a
*NUP133*	c.2922T>G	p.Ser974Arg	Steroid-resistant nephrotic syndrome (SRNS)	Yes (CRISPR/Cas9-edited human podocytes, transcriptome analysis, cytoskeleton analysis)	LP(PS3+PM2+PP3+PP4)	Rogg et al., 2022
*MTX2*	c.95delA	p.Q32Qfs*26	Nephrotic proteinuria, glomerulopathy	Yes (Pod-Mtx2-KO mice, podocyte assays)	P(PVS1+PS3+PM2+PP4)	Li et al., 2024a
*MTX2*	c.378 + 1G>A	Splice-site variant	Nephrotic proteinuria, glomerulopathy	Yes	P(PVS1+PS3+PM2+PP4)	Li et al., 2024b
*MTX2*	c.500_501insT	p.R167Rfs*2	SRNS, multisystem disorder	Yes (mouse model, mitochondrial studies)	P(PVS1+PS3+PM2+PP4)	Li et al., 2024c
*COQ8B*	c.1027C>T	p.Arg343Trp	Steroid-resistant nephrotic syndrome (SRNS)	NO	VUS(PM2+PP3+PP4)	Vazquez Fonseca et al., 2017
*CD151*	c.631A>T	p.Lys211Ter	Nephrotic-range proteinuria; microscopic haematuria; GBM abnormalities	Yes (patient kidney biopsy, red blood cell serology, zebrafish)	LP(PS3+PM2+PP3+PP4)	Naylor et al., 2022
*CRB2*	c.3106_3107del	p.Gly1036Alafs*43	Early-onset SRNS/FSGS	Yes (CRB2 deficiency podocyte model)	P(PVS1+PS3+PM2+PP4)	Sun et al., 2025
*CRB2*	c.3221_3229del	p.Leu1074_Asp1076del	SRNS/FSGS	Yes (CRB2 knockdown/mechanotransduction assays)	P(PVS1+PS3+PM2+PP4)	Sun et al., 2025
*FAT1*	c.5648T>A	p.Leu1883Ter	Proteinuria; kidney hypodysplasia; ESKD	Yes (patient-derived urinary epithelial cells)	​	Fabretti et al., 2021
*FAT1*	c.8446_8447dupGC	p.Phe2817HisfsTer13	Proteinuria; glomerular hypertrophy; CKD	Yes (patient-derived urinary epithelial cells)	P(PVS1+PS3+PM2+PP4)	Fabretti et al., 2021
*FAT1*	c.2563G>A	p.Gly855Arg	Diffuse mesangial sclerosis; nephrotic-range proteinuria	Yes (patient-derived urinary epithelial cells)	LP(PS3+PM2+PP3+PP4)	Fabretti et al., 2021
*FAT1*	c.5539G>A	p.Val1847Ile	Diffuse mesangial sclerosis; nephrotic-range proteinuria	Yes (patient-derived urinary epithelial cells)	LP(PS3+PM2+PP3+PP4)	Fabretti et al., 2021
*CD2AP*	c.592_593insTCAC	p.Ser198IlefsTer3	Childhood-onset FSGS; proteinuria; progression to ESKD	Yes (CRISPR/Cas9 knock-in mice)	P(PVS1+PS3+PM2+PP4)	Takano et al., 2019

#### 2.2.5 Network-level supporting evidence

Beyond single-gene analyses, protein interaction studies and functional investigations further underscore the central role of Rho-GTPase signaling networks in podocyte biology. Regulators such as CdGAP have been shown to modulate focal adhesion dynamics and filtration barrier function (Matsuda et al., 2022). Recent genetic and functional studies indicate that many podocytopathy-associated genes regulate cytoskeletal organization through signaling pathways, vesicular trafficking, or organelle homeostasis rather than acting as structural components (Majmundar et al., 2021; Schneider et al., 2020; Li et al., 2021; Matsuda et al., 2022).

Taken together, these findings indicate a gradual shift from a model centered on structural protein defects toward one that emphasizes dysregulation within cytoskeletal regulatory networks.

### 2.3 Mechanistic framework of cytoskeletal homeostasis

Integration of the evidence outlined above suggests that cytoskeletal dysfunction in podocytes may be better understood as a disturbance of dynamic regulatory processes rather than a consequence of isolated structural abnormalities. In contrast to earlier models focused on proteins such as ACTN4 and INF2, recent studies highlight the role of upstream signaling pathways in shaping cytoskeletal behavior.

Alterations in genes such as *NOS1AP* point to disrupted Rho-GTPase signaling as a potential initiating event (Majmundar et al., 2021), while findings related to *NOS1AP-C1orf226* indicate that transcript-level regulation may also contribute, although broader applicability remains uncertain (Buerger et al., 2025). Observations involving *DAAM2* and *MYO9A* further suggest that cytoskeletal regulation involves a complex network of interacting nodes rather than a linear pathway (Schneider et al., 2020; Li et al., 2021).

At the same time, studies on *TBC1D8B* raise the possibility that cytoskeletal organization is functionally coupled to membrane trafficking processes. By influencing nephrin endocytosis and recycling, alterations in this pathway may indirectly reshape cytoskeletal architecture, although the precise mechanisms have yet to be fully defined (Kampf et al., 2019; Milosavljevic et al., 2022; Jones et al., 2006). More broadly, endocytic and vesicular trafficking pathways provide a mechanism through which membrane protein distribution can influence cytoskeletal remodeling. By controlling the localization and turnover of slit diaphragm components, trafficking-associated genes may regulate the availability of signaling platforms required for actin dynamics and podocyte adaptation.

In this context, cytoskeletal homeostasis can be viewed as emerging from the interplay of multiple regulatory layers, including upstream signaling pathways, actin assembly dynamics, and membrane-associated processes. Rather than serving solely as a structural scaffold, the cytoskeleton appears to function as an integrative platform that translates diverse perturbations into functional changes at the level of the filtration barrier.

It should be noted that this framework is largely derived from recent genetic and experimental studies, and its general applicability across different forms of proteinuric disease remains to be established.

## 3 Membrane protein trafficking and slit diaphragm dynamics

### 3.1 Evolution of the slit diaphragm concept: from a structural barrier to a dynamic molecular assembly network

The slit diaphragm (SD) has traditionally been regarded as a specialized intercellular junction formed between adjacent podocyte foot processes and an essential component of the glomerular filtration barrier. Classical genetic studies established that mutations affecting core SD proteins, including nephrin encoded by *NPHS1* (Kestilä et al., 1998) and podocin encoded by *NPHS2* (Boute et al., 2000), cause severe hereditary proteinuric disorders, highlighting the essential role of these proteins in maintaining filtration barrier integrity.

However, recent molecular and structural studies have substantially expanded this classical concept. Rather than functioning as isolated structural components, SD proteins constitute a dynamic molecular network involving membrane organization, protein assembly, intracellular signaling, and cytoskeletal regulation. The integrity of the SD depends not only on the presence of individual proteins but also on their coordinated interactions and higher-order organization. Accordingly, the molecular basis of SD homeostasis is better understood as an interconnected architecture in which membrane proteins, adaptor molecules, signaling components, and cytoskeletal regulators function cooperatively.

The central SD molecular axis consists of the nephrin–NEPH1/KIRREL1–podocin–CD2AP complex (Donoviel et al., 2001). Nephrin and podocin cooperate to establish specialized membrane domains at the slit diaphragm, while NEPH1/KIRREL1 contributes to the assembly and stabilization of the SD complex. On the cytoplasmic side, CD2AP acts as an adaptor and scaffold molecule that connects SD-associated proteins with intracellular signaling pathways and the actin cytoskeleton (Shih et al., 1999). Through phosphorylation-dependent recruitment of NCK1/NCK2 adaptor proteins, nephrin couples extracellular filtration stress signals to actin polymerization machinery, thereby maintaining podocyte foot process architecture (Jones et al., 2006).

MAGI2 participates in higher-order SD assembly and nephrin organization through multivalent interactions (Zhang et al., 2021). Biallelic *MAGI2* variants cause congenital/early-onset nephrotic syndrome, while podocyte-specific *Magi2* loss causes albuminuria and glomerulosclerosis (Bierzynska et al., 2017; Shirata et al., 2017). Functional studies further implicate Rho-like GTPase regulation, particularly MAGI2–RapGEF2–Rap1 signaling, in cytoskeletal injury, while *magi2a*-deficient zebrafish exhibit corticosteroid-exacerbated nephrotic injury (Ashraf et al., 2018; Zhu B. et al., 2019; Jobst-Schwan et al., 2019). Together, these findings position MAGI2 at the interface of SD organization and cytoskeletal signaling within the molecular network summarized in Figure 2.

**FIGURE 2:**
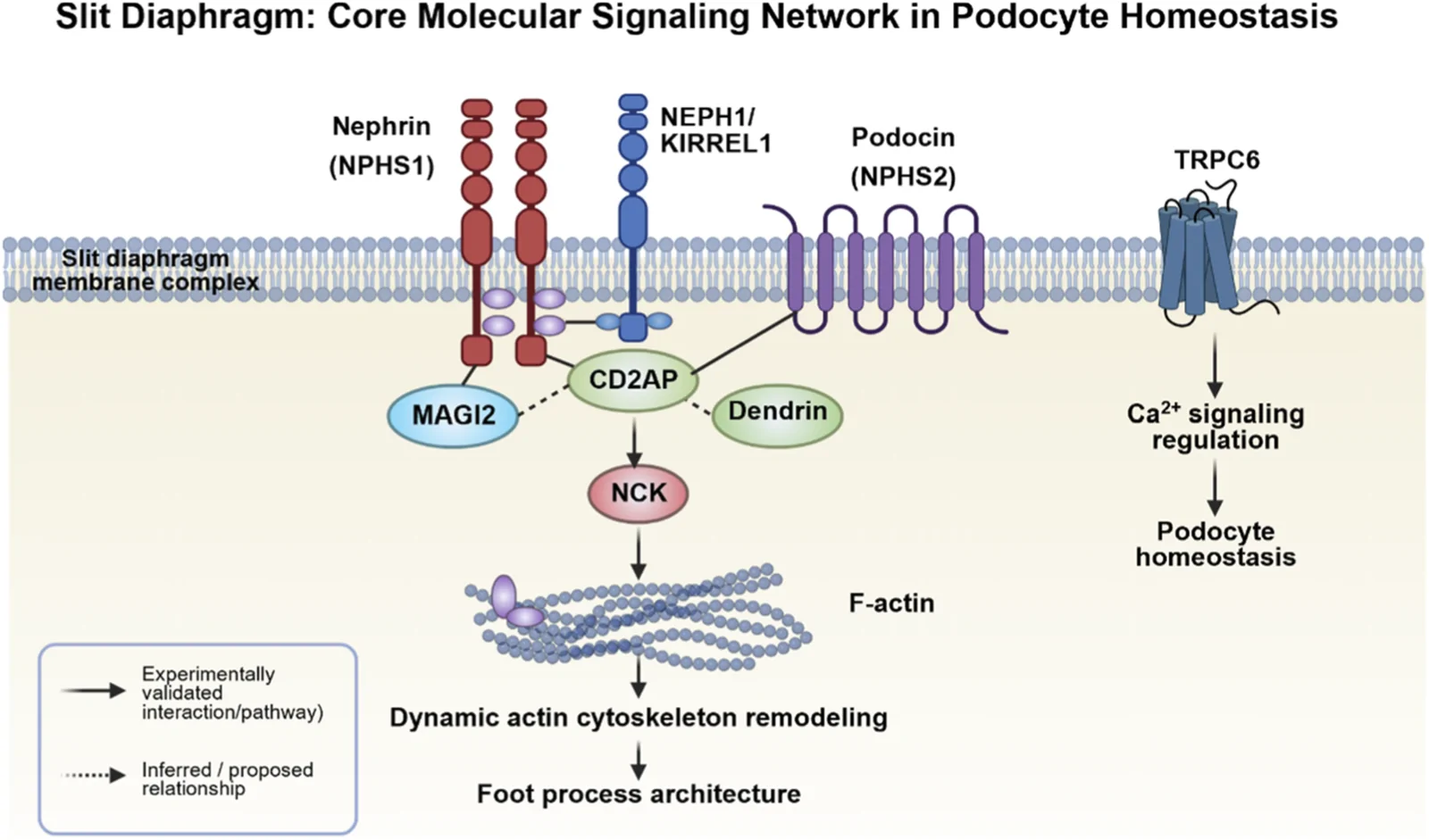
Core molecular signaling network of the slit diaphragm in podocyte homeostasis. The slit diaphragm (SD) functions as a dynamic molecular assembly that integrates membrane-associated proteins, intracellular adaptor molecules, and cytoskeletal regulators to maintain podocyte structural integrity. The nephrin (*NPHS1*)–NEPH1/KIRREL1–podocin (*NPHS2*) complex forms the core membrane architecture of the SD, while CD2AP acts as an adaptor linking SD-associated proteins to intracellular signaling pathways and the actin cytoskeleton. Through recruitment of NCK proteins, SD signaling regulates actin cytoskeleton remodeling and maintenance of foot process architecture. MAGI2 and Dendrin participate in the organization of the SD-associated protein interaction network. TRPC6-mediated calcium signaling represents an additional regulatory pathway involved in podocyte homeostasis. Solid arrows indicate experimentally validated interactions or signaling pathways.

CD2AP is an essential slit-diaphragm adaptor, as demonstrated by severe nephrotic disease in *Cd2ap*-null mice and its coordinated expression with nephrin during podocyte development (Li et al., 2000; Shih et al., 2001; Shih et al., 1999). *CD2AP* haploinsufficiency increases susceptibility to glomerular injury, while heterozygous variants have been identified in human sporadic nephrotic syndrome/FSGS (Kim et al., 2003; Gigante et al., 2009; Tsvetkov et al., 2016). More definitive evidence came from a homozygous *CD2AP* frameshift mutation causing FSGS in siblings and recapitulating glomerular disease in gene-edited mice (Takano et al., 2019). Therefore, *CD2AP* may contribute not only to the physical coupling of slit diaphragm components with the actin cytoskeleton but also to the spatial organization and functional coordination of the broader SD molecular network.

Recent biophysical studies have provided further insights into the structural basis underlying *CD2AP* function. Biophysical characterization of full-length human *CD2AP* demonstrated a predominantly disordered secondary structure and the capacity to form higher-order homo-oligomeric assemblies, suggesting that conformational flexibility and oligomeric organization may contribute to the higher-order assembly of molecular complexes at the slit diaphragm (Qadri et al., 2024). Recent structural syntheses have further emphasized that intrinsically disordered regions and oligomerization may contribute to the organization of higher-order protein–protein networks within the slit diaphragm (Qadri et al., 2026a). These findings provide a structural basis for considering how alterations in CD2AP conformation and oligomeric organization may influence higher-order SD network assembly beyond simple changes in protein abundance, although the functional significance of these oligomeric states in podocytes requires further investigation.

Extending these observations, an emerging preprint study investigating the disease-associated *CD2AP* K301 M variant provides a representative example of how a single genetic alteration may propagate through multiple molecular layers within the slit diaphragm network. Using complementary structural, biophysical, and proteomic approaches, Qadri et al. compared wild-type and K301 M *CD2AP* and reported alterations in higher-order oligomeric assembly and structural organization. Compared with wild-type *CD2AP*, the K301 M variant showed reduced oligomerization capacity and was also associated with altered interaction with podocin and remodeling of *CD2AP*-associated protein interaction networks. These findings suggest that disease-associated *CD2AP* variants may compromise slit diaphragm integrity through mechanisms extending beyond changes in protein abundance, including disruption of scaffold assembly, partner recognition, and higher-order molecular organization. Because this study is currently available only as a non-peer-reviewed preprint, these findings should be interpreted cautiously and require further independent experimental validation. Nevertheless, this emerging study provides a mechanistically relevant example illustrating the proposed genotype–protein assembly–cellular phenotype framework (Qadri et al., 2026b).

*TRPC6* (OMIM: 603652), a calcium-permeable SD-associated channel, was first genetically linked to familial FSGS through pathogenic mutations and connects SD integrity with calcium-dependent signaling pathways (Reiser et al., 2005; Winn et al., 2005). Moreover, increased wild-type *TRPC6* expression occurs in acquired proteinuric diseases, and podocyte-specific overexpression of either wild-type or mutant TRPC6 is sufficient to induce glomerular disease in mice (Möller et al., 2007; Krall et al., 2010). Recent electrophysiological analyses further identify delayed channel inactivation as a common feature of FSGS-associated *TRPC6* variants, refining the conventional gain-of-function model toward prolonged abnormal cation signaling (Heeringa et al., 2009; Okada et al., 2025).

Taken together, available genetic, structural, and functional evidence indicates that disease-associated variants in SD proteins may exert their pathogenic effects through mechanisms extending beyond simple loss of protein abundance. Such variants can alter protein folding, physicochemical properties, oligomerization, intracellular localization, or interactions with molecular partners, thereby perturbing higher-order molecular organization and the stability of the SD complex (Nishibori et al., 2004; Schwarz et al., 2001). From this perspective, the progression from genotype to podocyte injury can be conceptualized as a hierarchical process in which variant-induced changes in protein properties impair molecular assembly and protein–protein interactions, destabilize the SD network, and subsequently disturb cytoskeletal and signaling homeostasis, ultimately contributing to podocyte injury and proteinuria (Jones et al., 2006; Nishibori et al., 2004; Schwarz et al., 2001).

Experimental evidence further supports a direct role of podocin in higher-order SD organization. Podocin forms oligomeric assemblies within membrane microdomains and interacts with nephrin and *CD2AP* (Schwarz et al., 2001), while recent evidence indicates that podocin oligomerization contributes to the ordered organization of nephrin chains (Antal-Kónya et al., 2026). Thus, disease-associated *NPHS2* variants that perturb membrane association, oligomerization, or partner interactions could destabilize SD assembly even in the absence of marked reductions in total protein abundance. A recent non-peer-reviewed computational preprint by Gajula et al. further supports the possibility that disease-associated variants in core slit diaphragm proteins may alter intrinsic protein properties beyond affecting localization or degradation. The authors analyzed nonsynonymous variants in several SD proteins, including podocin, nephrin, *CD2AP, NEPH1,* and *TRPC6,* and predicted mutation-associated changes in protein stability, aggregation propensity, solubility, and surface properties. These predictions suggest that genetic variants may influence SD integrity through altered protein physicochemical characteristics and molecular interactions. However, as these observations are based on computational predictions, further experimental validation is required (Gajula et al., 2026).

Within the multi-layer framework proposed in this review, the slit diaphragm represents a critical integration hub connecting membrane trafficking, cytoskeletal homeostasis, and extracellular mechanical sensing. Disturbance of SD organization may therefore propagate across multiple cellular compartments, explaining how genetically diverse podocytopathy-associated variants converge on common pathological outcomes. Thus, the SD can be viewed not as an isolated structural layer, but as a molecular interface through which membrane organization, intracellular signaling, cytoskeletal remodeling, and mechanical inputs are integrated within the broader podocyte homeostatic network.

### 3.2 Recent advances and emerging evidence

#### 3.2.1 Mechanistic reinterpretation of classical genes

New experimental approaches have led to a revised understanding of the pathogenic mechanisms associated with classical slit diaphragm genes. Studies using patient-derived iPSC kidney organoids have shown that certain *NPHS1* variants do not simply reduce nephrin expression but interfere with its trafficking and membrane localization, preventing proper assembly of slit diaphragm precursors (Ohmori et al., 2021).

Comparable observations have been reported for *NPHS2*. Allelic series analyses indicate that missense variants in podocin may affect protein stability, disrupt interactions with nephrin, or alter intracellular distribution (Dorison et al., 2023). In particular, the R138Q variant has been shown to form aberrant complexes with keratin-8, resulting in impaired trafficking to the plasma membrane. Partial restoration of correct localization has been achieved through the use of small-molecule chaperones (Kuzmuk et al., 2024).

A systematic comparison of multiple disease-associated podocin variants, including G92C, V180M, R138Q, R238S, and R291W, has further demonstrated that these variants share reduced plasma membrane abundance, while R138Q exhibits a distinct susceptibility to ubiquitin–proteasome–dependent degradation that can be partially reversed by proteasome inhibition (Lu et al., 2026).

These findings indicate that *NPHS2*-associated disease may arise from heterogeneous defects in protein trafficking and degradation, supporting the concept that distinct molecular mechanisms can underlie variants within the same gene. Beyond defects in intracellular trafficking and degradation, disease-associated podocin variants may also affect higher-order organization of the slit diaphragm complex through altered protein conformation, membrane association, oligomerization behavior, and intermolecular interactions.

#### 3.2.2 Nephrin turnover and trafficking defects

Further studies have demonstrated the consequences of impaired TBC1D8B-mediated nephrin trafficking. Loss of *TBC1D8B* function results in mislocalization of nephrin within intracellular compartments, leading to progressive destabilization of the slit diaphragm. These observations imply that nephrin is not statically anchored but undergoes continuous cycling between membrane and endosomal pools. Disruption of this cycle may gradually reduce functional protein availability at the filtration interface (Milosavljevic et al., 2022; Dorval et al., 2019; Lang et al., 2022).

Although the involvement of Rab11-dependent trafficking pathways is supported by experimental data, the precise regulatory mechanisms in human podocytes remain incompletely defined and likely involve additional interacting components (Kampf et al., 2019; Milosavljevic et al., 2022).

The clinical spectrum of *TBC1D8B*-associated disease has recently been expanded by the identification of a pathogenic frameshift variant affecting the TBC domain in a middle-aged individual with FSGS, representing a late-onset presentation attributed to this gene (Hu et al., 2024). This observation indicates that impaired nephrin trafficking due to *TBC1D8B* deficiency may manifest beyond early-onset nephrotic syndrome, supporting the concept that disruption of slit diaphragm turnover can contribute to disease across a broad age range.

#### 3.2.3 Non-genetic and cross-system influences

Evidence from non-genetic studies further supports the concept that slit diaphragm stability depends on dynamic regulation. Autoantibodies targeting nephrin have recently been identified in podocytopathies, supporting the concept that slit diaphragm dysfunction can arise through immune-mediated disruption of nephrin even in the absence of pathogenic germline variants (Hengel et al., 2024). These observations indicate that membrane protein function can be perturbed through immune-mediated mechanisms in the absence of underlying genetic mutations.

In parallel, studies of nucleoporin genes have suggested indirect links between nuclear regulation and slit diaphragm integrity. For example, alterations in *NUP160* have been associated with changes in transcriptional programs and subsequent effects on protein localization within podocytes (Li Y. et al., 2024). Although these findings do not directly implicate vesicular trafficking machinery, they suggest that upstream regulatory disturbances may influence membrane protein dynamics.

#### 3.2.4 Supporting evidence from vesicular transport systems

Additional support for the role of membrane trafficking comes from studies of vesicle delivery systems. Components of the exocyst complex, including *EXOC4* (OMIM: 608185) and *EXOC5* (OMIM: 609412), are involved in targeting vesicles to specific regions of the plasma membrane. Disruption of this complex has been shown to impair the localization of nephrin and Neph1, resulting in structural abnormalities of foot processes (Nihalani et al., 2019).

Most of these observations were reported prior to the recent expansion of genetic studies; nevertheless, they provide a mechanistic background indicating that directed vesicle transport contributes to the maintenance of slit diaphragm architecture.

### 3.3 Mechanistic framework of slit diaphragm dynamics

Taken together, available evidence supports a reinterpretation of slit diaphragm function in terms of dynamic homeostasis rather than static structure. Stability of the nephrin–podocin complex appears to depend not only on protein expression but also on a series of interconnected processes, including folding and quality control, vesicular transport, endocytosis, and recycling (Kampf et al., 2019; Milosavljevic et al., 2022; Ohmori et al., 2021; Dorison et al., 2023; Hengel et al., 2024).

Within this framework, perturbations at different stages may produce similar functional outcomes. Impaired folding or trafficking can reduce membrane localization of nephrin; alterations in endosomal sorting may lead to intracellular accumulation; defects in recycling can limit replenishment of surface pools. These processes do not operate in isolation, and disruption at one level may influence others over time.

Findings related to *TBC1D8B*, nephrin autoantibodies, and nucleoporin-associated regulation suggest that diverse upstream disturbances may converge on a common consequence, namely, altered abundance or distribution of slit diaphragm proteins (Milosavljevic et al., 2022; Li Y. et al., 2024; Hengel et al., 2024). Direct molecular convergence of these pathways has not been demonstrated, and current understanding remains largely based on functional parallels rather than shared signaling cascades.

From this perspective, proteinuria can be interpreted as a consequence of failure in maintaining membrane protein homeostasis at the slit diaphragm. Rather than an abrupt structural breakdown, the process may involve progressive destabilization driven by impaired renewal and redistribution of key components.

As with other emerging models, the extent to which this framework applies across different forms of proteinuric disease requires further investigation.

## 4 Nuclear, transcriptional, and developmental regulation

### 4.1 Conventional perspective

Interpretation of podocyte injury has traditionally focused on cytoplasmic structures, particularly the slit diaphragm, actin cytoskeleton, mitochondrial function, and lysosomal pathways (Pavenstädt et al., 2003; Shankland, 2006; Hartleben et al., 2010). In contrast, nuclear processes, including transcriptional regulation, nuclear transport, and nuclear pore complex integrity, have not been widely considered central to the development of proteinuria, although emerging evidence suggests that alterations in these nuclear mechanisms may contribute to podocyte dysfunction and glomerular disease (Rogg et al., 2022; Chugh, 2007; Braun et al., 2016).

This view has begun to shift with the accumulation of genetic and functional evidence. Several recent studies suggest that alterations in nuclear organization and gene regulatory mechanisms may influence podocyte behavior and contribute to disease progression (Weng et al., 2021; Rogg et al., 2022; Han et al., 2024; Li Y. et al., 2024). These observations suggest that podocyte dysfunction may arise not only from structural defects at the cell periphery but also from disturbances in transcriptional regulation, developmental programs, and nuclear organization.

### 4.2 Podocyte transcriptional and developmental regulation

#### 4.2.1 Classical transcriptional regulators: WT1 and LMX1B

Transcriptional regulation is essential for both podocyte differentiation and maintenance of mature podocyte identity. WT1 represents a central regulator of podocyte-specific gene expression and controls several genes involved in filtration barrier formation and maintenance, including *NPHS2, MAFB,* and *MAGI2* (Dong et al., 2015). Similarly, LMX1B contributes to podocyte differentiation and maintenance of foot process architecture by regulating transcriptional programs required for cytoskeletal organization and filtration barrier integrity (Burghardt et al., 2013). These established transcription factors demonstrate that podocyte dysfunction may result not only from defects in structural proteins but also from disruption of gene regulatory programs.

#### 4.2.2 Emerging developmental mechanism: SMAD2

Recent studies have extended this concept to developmental regulators. Using engineered human induced pluripotent stem cell models, Bhattacharya et al. demonstrated that pathogenic *SMAD2* variants disrupt mesodermal and intermediate mesodermal patterning, leading to impaired podocyte differentiation, defective foot process development, and compromised glomerular filtration barrier function (Bhattacharya et al., 2025). These findings suggest that genetic podocytopathies may arise not only from impaired maintenance of mature podocytes but also from disturbances in developmental programs required to establish functional podocyte identity.

### 4.3 Nuclear regulatory and nucleoporin-associated mechanisms

#### 4.3.1 Nuclear regulatory proteins: *TRIM8*-associated phenotypes

*TRIM8* has emerged as a representative example of nuclear regulatory involvement in podocyte-related disease. *De novo* truncating variants in *TRIM8* have been identified in patients with a syndromic form of FSGS accompanied by developmental delay and epilepsy. Functional studies indicate that wild-type *TRIM8* localizes to nuclear bodies in both podocytes and neurons, whereas truncated variants exhibit a more diffuse distribution within the nucleoplasm and cytoplasm, consistent with altered nuclear organization (Weng et al., 2021).

These findings suggest that disruption of nuclear protein localization and associated transcriptional responses may contribute to podocyte dysfunction. It should be noted, however, that *TRIM8*-associated conditions typically involve multisystem manifestations, and the renal phenotype may represent one component of a broader developmental disorder (Weng et al., 2021). As such, *TRIM8* is best considered as indicative of nuclear regulatory involvement rather than a classical podocyte-specific disease gene.

#### 4.3.2 Nucleoporin genes with functional support: *NUP133*

Among nuclear pore complex components, *NUP133* (OMIM: 607613) provides relatively strong functional evidence linking nuclear architecture to podocyte biology. Loss-of-function models generated using CRISPR/Cas9 in human podocytes have demonstrated that *NUP133* deficiency disrupts nuclear pore assembly, alters transcriptional profiles, and impairs cytoskeletal organization and cellular morphology (Rogg et al., 2022).

These observations suggest that nucleoporins may influence podocyte function beyond their structural role in nuclear transport. By modulating transcriptional programs and possibly coordinating signaling pathways that regulate the cytoskeleton, *NUP133* may serve as a point of interaction between nuclear processes and cellular architecture.

#### 4.3.3 Clinically associated nucleoporin genes: *NUP93, NUP107*, and *NUP160*

Additional nucleoporin genes have been implicated through clinical studies. Biallelic variants in *NUP93* (OMIM: 614351), *NUP107* (OMIM: 607617), and *NUP160* (OMIM: 607614) have been identified in early-onset steroid-resistant nephrotic syndrome and are often associated with rapid progression to end-stage kidney disease (Han et al., 2024). These findings support the view that disruption of nuclear pore complex components constitutes a distinct category within genetic podocytopathies.

Experimental models provide further insight into these mechanisms. Podocyte-specific deletion of *NUP160* in mice results in proteinuria, foot process effacement, and glomerulosclerosis (Li Y. et al., 2024). Podocyte-specific *NUP160* deletion leads to selective reduction of CDC42 protein without corresponding transcript changes, accompanied by decreased CDC42 activity and progressive proteinuria (Liu et al., 2025). These findings provide functional evidence linking nuclear pore complex integrity to Rho-GTPase–mediated cytoskeletal regulation in podocytes.

Despite these advances, the relationship between specific nucleoporin variants and clinical phenotypes remains incompletely defined. Differences in disease severity and progression across genes suggest that multiple mechanisms may be involved. Recent clinical data further expand this spectrum by describing genotype–phenotype associations in steroid-resistant nephrotic syndrome with nuclear pore gene mutations, reinforcing the view that nucleoporin-associated disease represents a clinically relevant subgroup of genetic proteinuric kidney disease (Yang et al., 2025).

#### 4.3.4 Network-level observations: nucleoporin-associated disease spectrum

Mutations in multiple nucleoporin genes, including *NUP93, NUP107, NUP133,* and *NUP160*, have been identified in patients with steroid-resistant nephrotic syndrome, suggesting that disruption of nuclear pore complex components represents a recurring disease mechanism (Rogg et al., 2022; Han et al., 2024; Li Y. et al., 2024; Liu et al., 2025). This pattern suggests that disruption of the nuclear pore complex is not an isolated phenomenon but may represent a recurring mechanism within a subset of genetic kidney diseases.

At the same time, considerable heterogeneity exists within this group. Different nucleoporins may affect distinct aspects of nuclear function, including pore assembly, nuclear transport, transcriptional regulation, and cell cycle control (Rogg et al., 2022; Li Y. et al., 2024; Liu et al., 2025). Consequently, it is more appropriate to consider nucleoporin-associated podocytopathy as a functional category rather than a single mechanistic pathway.

### 4.4 Integrated framework for nuclear, transcriptional, and developmental regulation

The evidence outlined above supports an expanded view of nuclear and transcriptional regulation in podocyte biology. Classical transcription factors such as WT1 and LMX1B contribute to the establishment and maintenance of podocyte-specific gene expression programs, whereas developmental regulators such as SMAD2 influence podocyte specification and differentiation. In parallel, TRIM8 and nucleoporin-associated mechanisms illustrate how alterations in nuclear organization and transport can disrupt transcriptional responses and downstream cellular processes.

Within this framework, disturbances in transcriptional regulation, developmental specification, nuclear organization, or nuclear transport may influence cytoskeletal dynamics, membrane protein distribution, and cellular morphology. These effects suggest that nuclear and transcriptional abnormalities may function as upstream perturbations that propagate across multiple components of podocyte homeostasis.

Observations from *TRIM8* and nucleoporin-associated studies suggest that podocyte injury may, in some cases, originate from nuclear-level perturbations. This interpretation differs from traditional models in which damage is assumed to arise at the level of the slit diaphragm or cytoskeleton. Instead, nuclear dysfunction may act as an upstream event that gradually affects multiple aspects of podocyte regulation (Weng et al., 2021; Rogg et al., 2022; Braun et al., 2016).

It remains unclear to what extent these mechanisms contribute to more common forms of proteinuric disease. Much of the current evidence is derived from genetic disorders, cellular models, and animal studies. Further work will be required to determine how nuclear regulatory pathways interact with other components of podocyte homeostasis in human disease. Figure 3 summarizes a proposed model illustrating the coupling between nuclear regulation and mitochondrial function in podocyte homeostasis.

**FIGURE 3:**
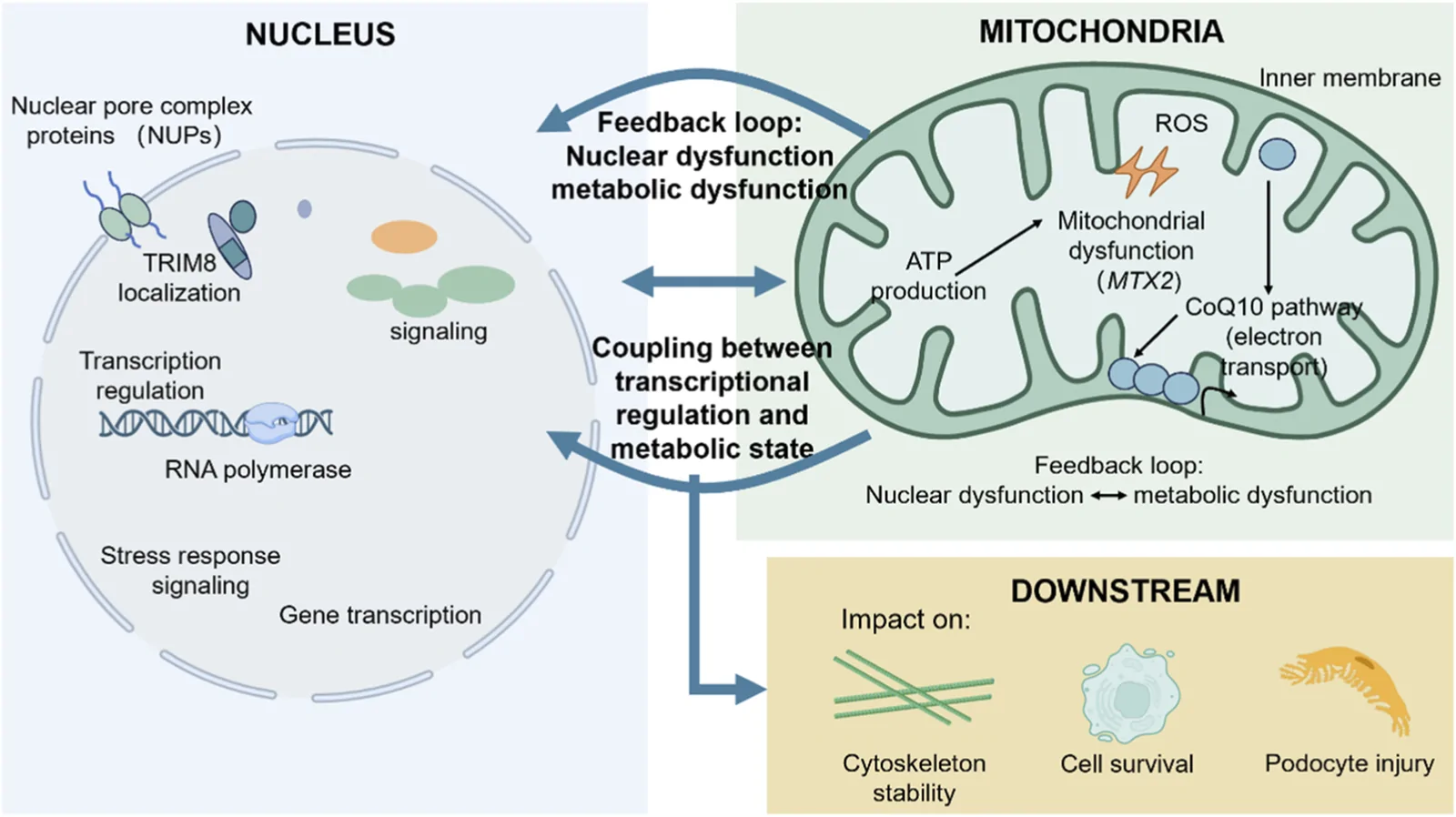
Coupling between nuclear regulation and mitochondrial function in podocyte homeostasis. Nuclear and mitochondrial processes are functionally interconnected in the regulation of podocyte stability. Nuclear pore complex (NUP) proteins and transcriptional regulators such as TRIM8 modulate gene expression programs and cellular stress responses. These nuclear signals influence mitochondrial function, including ATP production through oxidative phosphorylation and metabolic pathways such as the coenzyme Q10 (CoQ10) pathway. Conversely, mitochondrial dysfunction, characterized by altered energy production and increased reactive oxygen species (ROS), can feedback to affect nuclear transcriptional regulation. This bidirectional coupling integrates metabolic status with gene expression and contributes to cytoskeletal stability and cell survival. Disruption of this regulatory axis can amplify cellular stress responses and promote podocyte injury.

### 4.5 Genetic variants as drivers of protein-level network disruption

Although genetic studies have greatly expanded the spectrum of podocytopathy-associated genes, the pathogenic consequences of these variants cannot be fully explained by simple loss-of-function mechanisms. Increasing evidence indicates that disease-associated variants frequently influence protein-level properties, including folding stability, intracellular trafficking, membrane targeting, oligomerization, and interactions with molecular partners. These molecular alterations may subsequently disrupt higher-order protein assemblies and compromise podocyte homeostasis. Importantly, the consequences of disease-associated variants may extend beyond altered trafficking or localization. Variant-induced changes in intrinsic physicochemical properties, oligomerization, or interaction interfaces may impair higher-order molecular assembly even when total protein abundance is relatively preserved.

For example, mutations in *NPHS1*, encoding nephrin, may impair nephrin biosynthesis, maturation, and trafficking, resulting in reduced delivery of nephrin to the slit diaphragm and disruption of filtration barrier integrity (Li et al., 2001). Similarly, mutations in *NPHS2*, encoding podocin, can affect podocin processing and intracellular trafficking. Several disease-associated podocin variants exhibit defective plasma membrane targeting due to endoplasmic reticulum retention, demonstrating that altered protein localization represents an important mechanism underlying genetic podocytopathy (Roselli et al., 2010).

Beyond slit diaphragm components, mutations affecting cytoskeletal regulators further illustrate how genetic alterations propagate through molecular networks. Mutations in *ACTN4*, encoding α-actinin-4, alter actin-binding properties and disrupt cytoskeletal organization, leading to familial focal segmental glomerulosclerosis (Kaplan et al., 2000). Likewise, mutations in *INF2*, a formin protein involved in actin dynamics, impair cytoskeletal regulation and cause podocyte injury (Brown et al., 2010).

Therefore, genetic podocytopathies should be interpreted as disorders of molecular organization rather than isolated defects of individual genes. Genetic variants may initiate a cascade from altered protein properties to impaired molecular assembly, disrupted protein–protein interactions, network instability, and ultimately podocyte structural and functional failure.

## 5 Mitochondrial homeostasis and inter-organelle regulation

### 5.1 Conventional view of mitochondrial involvement

Podocytes are highly specialized cells that operate under sustained mechanical load and metabolic demand. Maintenance of foot process structure, cytoskeletal remodeling, and membrane trafficking all require continuous energy supply, which has traditionally placed mitochondrial function at the center of podocyte physiology.

Earlier studies largely interpreted mitochondrial dysfunction in terms of impaired bioenergetics. Mitochondrial dysfunction has been directly linked to podocyte injury, with experimental studies demonstrating that alterations in mitochondrial structure and respiratory chain organization contribute to proteinuria and glomerular damage (Li T. et al., 2024). Within this framework, genes involved in coenzyme Q10 biosynthesis, including *COQ8B* (OMIM: 615567) and *PDSS1* (OMIM: 607429), have been identified in patients with steroid-resistant nephrotic syndrome, supporting a role for mitochondrial metabolic pathways in disease pathogenesis (Habib et al., 2025; Zhang et al., 2024). Clinical observations indicating partial responsiveness to CoQ10 supplementation further supported the functional relevance of this pathway (Habib et al., 2025; Zhang et al., 2024).

While these findings remain important, they do not fully capture the range of mitochondrial functions implicated in recent studies.

### 5.2 Emerging evidence from recent studies

#### 5.2.1 Mitochondrial structural regulation: *MTX2*

Among recently identified genes, *MTX2* provides a direct link between mitochondrial integrity and podocyte function. Variants in *MTX2* have been associated with proteinuric kidney disease, and podocyte-specific deletion in murine models leads to albuminuria, foot process effacement, and glomerular injury (Li T. et al., 2024).

Functional analyses indicate that *MTX2* deficiency affects not only mitochondrial activity but also broader aspects of cellular behavior, including adhesion, migration, and endocytic capacity. Restoration of wild-type *MTX2* expression partially reverses these phenotypes. Mechanistically, loss of *MTX2* disrupts mitochondrial protein import and is accompanied by reduced levels of components within the Sam50–CHCHD3–Mitofilin axis, which is involved in maintaining cristae architecture (Li T. et al., 2024).

*APOL1* risk variants cause podocyte injury through multiple interconnected mechanisms: podocyte-specific G1/G2 expression induces kidney disease in mice, while lysosomal membrane injury and ER stress have been implicated in cellular models (Lan et al., 2014; Beckerman et al., 2017; Wen et al., 2018). Mitochondrial studies further demonstrate impaired function, mitochondrial translocation with permeability-transition pore opening, and altered lipid/energy homeostasis (Ma et al., 2017; Shah et al., 2019; Ge et al., 2021; Song et al., 2025). More recent work identifies APOL1-mediated monovalent cation transport at the plasma membrane as a proximal cytotoxic mechanism modulated by haplotype and surface localization (Datta et al., 2024; Gupta et al., 2024).

These observations suggest that mitochondrial structure, rather than energy production alone, may influence downstream cellular processes relevant to podocyte stability.

#### 5.2.2 Cristae organization and respiratory complex dynamics

Additional evidence has highlighted the importance of mitochondrial ultrastructure. Studies involving *NDUFS4* (OMIM: 602694) indicate that regulation of cristae morphology and respiratory chain organization may influence disease phenotypes. In experimental models of diabetic kidney disease, podocyte-specific overexpression of *NDUFS4* has been associated with improved mitochondrial structure and reduced proteinuria (Mise et al., 2024).

Interactions with cristae-associated proteins such as STOML2 further suggest that maintenance of inner mitochondrial membrane organization may contribute to functional stability (Mise et al., 2024). It should be noted that these observations are largely derived from acquired disease models and may not directly reflect mechanisms in monogenic podocytopathies.

#### 5.2.3 Inter-organelle communication: ER–mitochondria contact sites

Recent studies have also drawn attention to the role of communication between cellular organelles. Mitochondria-associated membranes (MAMs), which mediate contact between the endoplasmic reticulum and mitochondria, have been implicated in the regulation of calcium signaling, stress responses, and mitochondrial dynamics.

Under high-glucose conditions, downregulation of *MFN2* (OMIM: 608507) has been shown to disrupt MAM integrity, activate PERK signaling, and promote podocyte apoptosis (Cao et al., 2021). Additional work indicates that MAM-associated pathways can enhance mitochondrial fission through AKAP1–Drp1 signaling, further contributing to cellular injury (Li et al., 2023). Conversely, pharmacological interventions such as SGLT2 inhibition have been reported to restore aspects of MAM function and mitigate mitochondrial stress (Li X. et al., 2024; Zheng et al., 2025).

These findings suggest that mitochondrial dysfunction may, in part, arise from altered communication with other organelles rather than from intrinsic defects alone. However, most of this evidence originates from metabolic disease models, and its relevance to genetic podocytopathies remains to be clarified.

#### 5.2.4 Oxidative stress and quality control mechanisms

Mitochondrial oxidative stress continues to be recognized as an important factor in podocyte injury. Increased reactive oxygen species production and associated cellular stress responses have been observed in multiple experimental models of podocyte injury (Cao et al., 2021).

In addition, processes governing mitochondrial quality control, including fusion, fission, mitophagy, and biogenesis, contribute to maintaining cellular homeostasis under stress conditions (Dai et al., 2021). Disruption of these processes may lead to accumulation of dysfunctional mitochondria and progressive decline in podocyte function. These mechanisms, however, are broadly implicated across different forms of cellular stress and are not specific to individual genetic variants.

### 5.3 Mechanistic framework of mitochondrial integration

Taken together, recent findings support an expanded view of mitochondrial involvement in podocyte biology. Rather than functioning solely as energy-producing organelles, mitochondria appear to participate in multiple regulatory processes that influence cellular structure and signaling.

In this context, mitochondrial homeostasis can be understood as part of a broader system integrating metabolic status, redox balance, cytoskeletal dynamics, and membrane trafficking. Structural alterations, such as those observed with *MTX2* deficiency, may influence cellular behavior through mechanisms that extend beyond ATP production, as mitochondrial structural disruption can affect multiple aspects of podocyte homeostasis and function (Li T. et al., 2024). Similarly, changes in cristae organization or inter-organelle communication may affect signaling pathways that regulate podocyte adaptation to stress.

These processes are unlikely to operate independently. Perturbations in mitochondrial function may influence cytoskeletal organization, alter membrane protein turnover, or modify transcriptional responses. Conversely, disturbances in other cellular systems may secondarily affect mitochondrial stability. Although the precise interactions between these pathways remain incompletely defined, available evidence supports a model of functional interdependence.

From this perspective, mitochondrial dysfunction in proteinuric disease may reflect disruption of coordinated cellular regulation rather than a single primary defect. Further work will be required to determine how these mechanisms interact in human disease and whether specific components of this network can be selectively targeted.

## 6 GBM–podocyte interaction and mechanosensory regulation

### 6.1 Classical genetic framework of GBM-associated disease

The glomerular basement membrane (GBM) is positioned between endothelial cells and podocytes and constitutes a key structural component of the filtration barrier. Its composition is primarily defined by the collagen IV α3α4α5 network and associated laminin complexes. Variants in *COL4A3, COL4A4,* and *COL4A5* are well established as causes of Alport syndrome and related disorders, whereas mutations in *LAMB2* lead to Pierson syndrome (Zhu HT. et al., 2019; Qian et al., 2022; Zhang et al., 2023; Daga et al., 2025). These conditions often present with proteinuria and may progress to focal segmental glomerulosclerosis-like pathology.

Within this framework, the GBM has traditionally been viewed as a structural scaffold that provides mechanical support for podocyte attachment. Although this perspective explains many aspects of inherited GBM disorders, it does not fully account for how alterations in matrix composition influence podocyte behavior beyond structural integrity.

### 6.2 Emerging evidence from recent studies

#### 6.2.1 Genetic extension: regulatory variants and splicing alterations

Recent genetic studies have expanded the spectrum of GBM-related disease beyond coding mutations. Deep intronic variants in *COL4A5* have been shown to induce aberrant splicing and contribute to disease phenotypes that may be missed by conventional exon-focused sequencing approaches (Qian et al., 2022). Systematic analyses of *COL4A3–COL4A5* variants further indicate that splicing alterations represent an underrecognized mechanism in these disorders (Zhang et al., 2023).

In addition, noncanonical splice variants in *COL4A3* have been associated with milder, slowly progressive forms of Alport syndrome (Zhang et al., 2023). These findings suggest that alterations affecting transcript processing can influence GBM composition in ways that may not produce immediate structural disruption but could modify its functional properties over time.

#### 6.2.2 Structural and phenotypic correlation: Quantitative GBM alterations

Advances in imaging techniques have enabled more detailed characterization of GBM structure in disease. Quantitative analyses of collagen IV distribution have revealed measurable alterations in patients carrying Alport-associated variants, even in cases presenting with FSGS-like features (Puapatanakul et al., 2024).

Such observations indicate that GBM abnormalities may exist along a spectrum, ranging from subtle compositional changes to overt structural defects. These intermediate phenotypes provide a potential link between genetic variation and clinical presentation, suggesting that GBM alterations may influence disease progression in a graded manner. Recent studies in acquired kidney disease further support the concept that extracellular matrix remodeling can actively regulate podocyte function beyond genetic structural defects. Alterations in ECM composition, organization, and mechanical properties have been shown to influence podocyte behavior through mechanosensitive pathways, suggesting that changes in the extracellular environment may contribute to podocyte dysfunction by modifying cellular adaptation responses rather than solely by disrupting physical filtration support (Roye et al., 2024). In particular, changes in collagen IV composition may disrupt the GBM–podocyte interface and alter podocyte adhesion properties (Qian et al., 2022; Zhang et al., 2023; Daga et al., 2025). These mechanical alterations can further influence podocyte cytoskeletal organization through integrin-mediated mechanotransduction pathways. Activation of integrin-associated adhesion complexes can engage downstream pathways, including Rho GTPase-dependent cytoskeletal regulation and YAP/TAZ signaling, thereby influencing foot process morphology and podocyte adaptation to mechanical stress (Rinschen et al., 2017; Huynh-Cong et al., 2025; Saito et al., 2024).

#### 6.2.3 Cell–matrix interface regulation: adhesion complex integrity

Beyond GBM composition itself, the interface between podocytes and the extracellular matrix has emerged as an important determinant of cellular stability. *EPB41L5* (OMIM: 610972) has been shown to regulate extracellular matrix assembly and the recruitment of adhesion complex components, with loss of function leading to impaired podocyte attachment and structural instability (Maier et al., 2021).

Similarly, truncating variants in *CD151* (OMIM: 602243) have been associated with nephrotic-range proteinuria, GBM abnormalities, and foot process effacement (Naylor et al., 2022). *CD151* contributes to the organization of adhesion complexes linking podocytes to the underlying matrix. Disruption of these interactions may compromise the ability of podocytes to maintain stable attachment under mechanical stress.

*FAT1* (OMIM: 600976)-associated nephropathy further expands the spectrum of adhesion- and polarity-related genetic mechanisms. Novel *FAT1* mutations have been reported in patients with syndromic nephropathy and were linked to altered Hippo signaling, suggesting that podocyte adhesion defects may interact with transcriptional mechanosensory pathways (Fabretti et al., 2021).

These findings suggest that podocyte injury may arise not only from intrinsic cellular defects but also from altered properties of the cell–matrix interface.

#### 6.2.4 Mechanosensory signaling pathways

Recent studies further indicate that mechanosensory regulation in podocytes involves coordinated activity across multiple molecular components. Compound heterozygous *CRB2* (OMIM: 609720) mutations identified in familial SRNS/FSGS have been shown to induce YAP transcriptional activity and enhance mechanosignaling in a substrate stiffness–dependent manner (Sun et al., 2025).

In parallel, *EPB41L5* deficiency impairs mechanotransduction through the YAP/TAZ axis, while *ARHGAP29* (OMIM: 610496) has been identified as a downstream RhoGAP effector linking YAP/TAZ signaling to *RHOA* (OMIM: 165390)-dependent actin remodeling (Rogg et al., 2023). In addition, *CLDN5* (OMIM: 602101) contributes to adhesion complex stability through β1-integrin interactions (Wang et al., 2026), supporting a model in which multiple inputs converge on mechanosensory regulation at the GBM–podocyte interface.

Although these studies primarily derive from experimental models, they suggest that mechanical signaling is integrated with cytoskeletal and transcriptional regulation in maintaining podocyte function.

### 6.3 Mechanistic framework of mechanosensory regulation

Taken together, recent findings support a reinterpretation of the GBM as an active participant in podocyte regulation rather than a passive structural component. In this view, the GBM provides both physical support and a source of mechanical input that influences cellular behavior. This concept is supported not only by inherited GBM disorders but also by acquired renal diseases in which ECM remodeling alters the mechanical microenvironment experienced by podocytes.

Alterations in GBM composition, matrix assembly, or adhesion complex stability may change the mechanical environment experienced by podocytes. Such changes can be transmitted through integrin-based signaling pathways to the cytoskeleton and, subsequently, to transcriptional and metabolic processes. These responses are likely to be adaptive under physiological conditions but may become maladaptive when regulatory capacity is exceeded.

Importantly, these processes may not act independently. Changes at the GBM interface can influence cytoskeletal organization, which in turn affects membrane protein distribution and intracellular signaling. Conversely, defects in cytoskeletal or trafficking pathways may alter how mechanical signals are processed. This bidirectional relationship suggests that mechanosensory regulation is integrated within broader cellular networks rather than functioning as a separate pathway.

From this perspective, proteinuria associated with GBM abnormalities may reflect a combination of structural disruption and altered mechanical signaling. The relative contribution of these factors may vary across different disease contexts.

As with other emerging frameworks, the extent to which mechanosensory mechanisms contribute to human proteinuric disease remains to be fully defined. Further studies will be required to clarify how genetic variation, matrix composition, and cellular responses interact *in vivo*.

## 7 Reconstructing a multi-layer regulatory network of podocyte homeostasis

### 7.1 From structural disruption to coordinated regulation

Recent genetic findings have progressively altered the conceptual basis for interpreting podocyte injury in proteinuric diseases. Earlier models emphasized discrete structural defects, most prominently within the slit diaphragm or actin cytoskeleton. While these mechanisms remain relevant, they do not adequately explain why perturbations in genes with markedly different biological functions often lead to similar pathological outcomes. Single-cell transcriptomic studies further support this integrated view by showing that early proteinuric injury is accompanied by disrupted interactions among glomerular filtration barrier cells, indicating that podocyte dysfunction propagates beyond the injured cell itself (Clark et al., 2022).

An alternative interpretation emerges when these findings are considered collectively. Podocyte function appears to depend on continuous coordination across multiple cellular processes, rather than on the integrity of any single structural component. In physiological conditions, podocytes operate under persistent mechanical stress, metabolic demand, and fluctuating extracellular signals. Maintenance of the filtration barrier therefore requires ongoing adjustment across several interconnected layers, including cytoskeletal organization, membrane protein turnover, transcriptional regulation, and metabolic adaptation.

Within this context, proteinuria may be viewed less as a direct consequence of structural damage and more as the outcome of progressive imbalance across regulatory systems. This perspective provides a potential explanation for phenotypic convergence despite genetic heterogeneity.

### 7.2 Positioning emerging genes within a functional framework

Recent gene discoveries can be more readily interpreted when placed within a layered regulatory structure. Rather than representing isolated disease-associated loci, these genes appear to occupy distinct positions within a broader functional network.

At the interface between the podocyte and its extracellular environment, genes involved in cell–matrix interaction contribute to the interpretation of mechanical inputs. Disruption of components such as *EPB41L5* can impair matrix assembly and alter force transmission across the adhesion complex (Maier et al., 2021). Variants affecting collagen IV genes further suggest that changes in GBM composition may influence not only structural support but also the mechanical context sensed by podocytes (Qian et al., 2022; Zhang et al., 2023; Daga et al., 2025).

Cytoskeletal regulation constitutes a second layer in which signaling pathways translate external and internal cues into structural responses. Evidence from genes such as *NOS1AP* indicates that alterations in Rho-GTPase–dependent signaling can interfere with actin remodeling, thereby compromising foot process architecture (Majmundar et al., 2021). Additional candidates, including *MYO9A*, point to further complexity within this regulatory space, although their precise contributions remain to be clarified (Li et al., 2021).

Membrane trafficking pathways introduce another dimension of control by governing the localization and turnover of slit diaphragm components. *TBC1D8B* provides a well-characterized example, linking endosomal maturation and nephrin recycling to filtration barrier function (Kampf et al., 2019; Milosavljevic et al., 2022). Observations involving nephrin-targeting autoantibodies suggest that similar functional consequences can arise in the absence of genetic mutations, emphasizing the importance of membrane protein homeostasis (Hengel et al., 2024).

Nuclear regulation represents a more recently appreciated layer. Alterations in nucleoporin genes such as *NUP133* and *NUP160* have been associated with changes in transcriptional programs and cytoskeletal organization (Rogg et al., 2022; Li Y. et al., 2024; Liu et al., 2025). These findings raise the possibility that podocyte dysfunction may originate from disturbances in gene expression and nuclear transport, rather than solely from defects at the cell periphery.

At the level of intracellular energetics, mitochondrial pathways contribute to sustaining cellular function under stress. *MTX2* deficiency illustrates how disruption of mitochondrial structure can affect adhesion, migration, and membrane dynamics in addition to energy production (Li T. et al., 2024). Observations from acquired disease models further suggest that communication between organelles may influence these processes (Mise et al., 2024; Cao et al., 2021; Li et al., 2023; Li X. et al., 2024; Zheng et al., 2025), although direct genetic links remain limited.

At the network level, systematic BioID mapping of RHOA, RAC1, and CDC42 interactomes in human podocytes has identified 1,927 high-confidence protein–protein interactions, approximately half of which appear to be podocyte-enriched (Ibrahim et al., 2025). Among these, KIAA1522 was characterized as a CDC42-associated effector that contributes to foot process formation, with its depletion resulting in foot process effacement in zebrafish models (Ibrahim et al., 2025). These findings support the view that the podocyte cytoskeletal regulatory system represents a highly specialized network and provide a proteome-scale framework for interpreting how variants in diverse genes may converge on shared Rho GTPase–dependent mechanisms.

### 7.3 Integrated multi-layer network framework

Although genetic variants represent initiating events in many podocytopathies, their pathogenic consequences are often mediated through protein-level alterations rather than by gene disruption alone. Increasing evidence suggests that disease-associated variants exert their effects through alterations in protein stability, folding, localization, oligomerization, molecular interactions, and higher-order assembly (Li et al., 2001). These protein-level changes subsequently reshape cellular networks involving slit diaphragm organization, cytoskeletal dynamics, membrane trafficking, and stress responses, ultimately leading to podocyte dysfunction and proteinuria.

When considered together, these layers can be conceptualized as components of a continuous regulatory system. External mechanical cues are sensed at the GBM–podocyte interface, transmitted through adhesion complexes, and translated into cytoskeletal responses. These structural adjustments influence membrane protein distribution, which in turn affects signaling pathways and transcriptional programs. Mitochondrial activity supports these processes by maintaining metabolic balance under varying conditions.

Recent studies further indicate that these molecular layers are not independent modules but are interconnected through shared regulatory nodes and bidirectional feedback mechanisms. For example, the nephrin–NCK signaling axis provides a direct molecular link between slit diaphragm organization and actin cytoskeletal remodeling, allowing extracellular mechanical signals at the filtration barrier to be converted into intracellular structural responses (Nina et al., 2009). In parallel, membrane trafficking regulators such as *TBC1D8B* contribute to slit diaphragm maintenance by regulating nephrin trafficking, endosomal maturation, and membrane availability, thereby influencing filtration barrier function (Kampf et al., 2019; Milosavljevic et al., 2022).

Beyond membrane-associated regulation, nuclear and mitochondrial pathways also contribute to this interconnected network. Nuclear pore-associated proteins, including NUP133 and NUP160, influence podocyte homeostasis through regulation of transcriptional programs and cytoskeletal organization (Braun et al., 2016). Meanwhile, mitochondrial homeostasis provides metabolic support for maintaining energy-demanding processes, including actin remodeling, membrane trafficking, and stress adaptation (Coughlan et al., 2016). Therefore, disruption of one molecular layer may propagate across multiple cellular compartments, ultimately leading to progressive podocyte dysfunction.

Importantly, the proposed multi-layer network should be interpreted according to different levels of experimental evidence. Several molecular connections within the podocyte network have been directly demonstrated, including nephrin–NCK-mediated coupling between the slit diaphragm and actin cytoskeleton (Nina et al., 2009), CD2AP-associated interactions with slit diaphragm partners (Kampf et al., 2019; Shih et al., 1999; Schwarz et al., 2001), and TBC1D8B-dependent regulation of nephrin trafficking (Kampf et al., 2019). In contrast, some cross-layer relationships, such as the potential influence of mitochondrial dysfunction or nuclear regulatory pathways on slit diaphragm remodeling, remain mechanistically plausible but require further experimental validation.

Therefore, we distinguish experimentally validated molecular interactions from functional associations and conceptual hypotheses within the proposed framework. The slit diaphragm is positioned as a central integration hub because it represents an experimentally supported interface connecting membrane organization, cytoskeletal regulation, signaling pathways, and mechanical adaptation, whereas broader interactions involving metabolic and transcriptional regulation are presented as emerging or proposed connections (Grahammer et al., 2013).

Figure 4 summarizes this evidence-stratified network architecture and illustrates how the slit diaphragm serves as a central point of convergence among multiple regulatory layers. By positioning experimentally supported interactions alongside proposed cross-layer connections, the schematic emphasizes both the functional integration of podocyte homeostasis and the different levels of evidence underlying individual network relationships.

**FIGURE 4:**
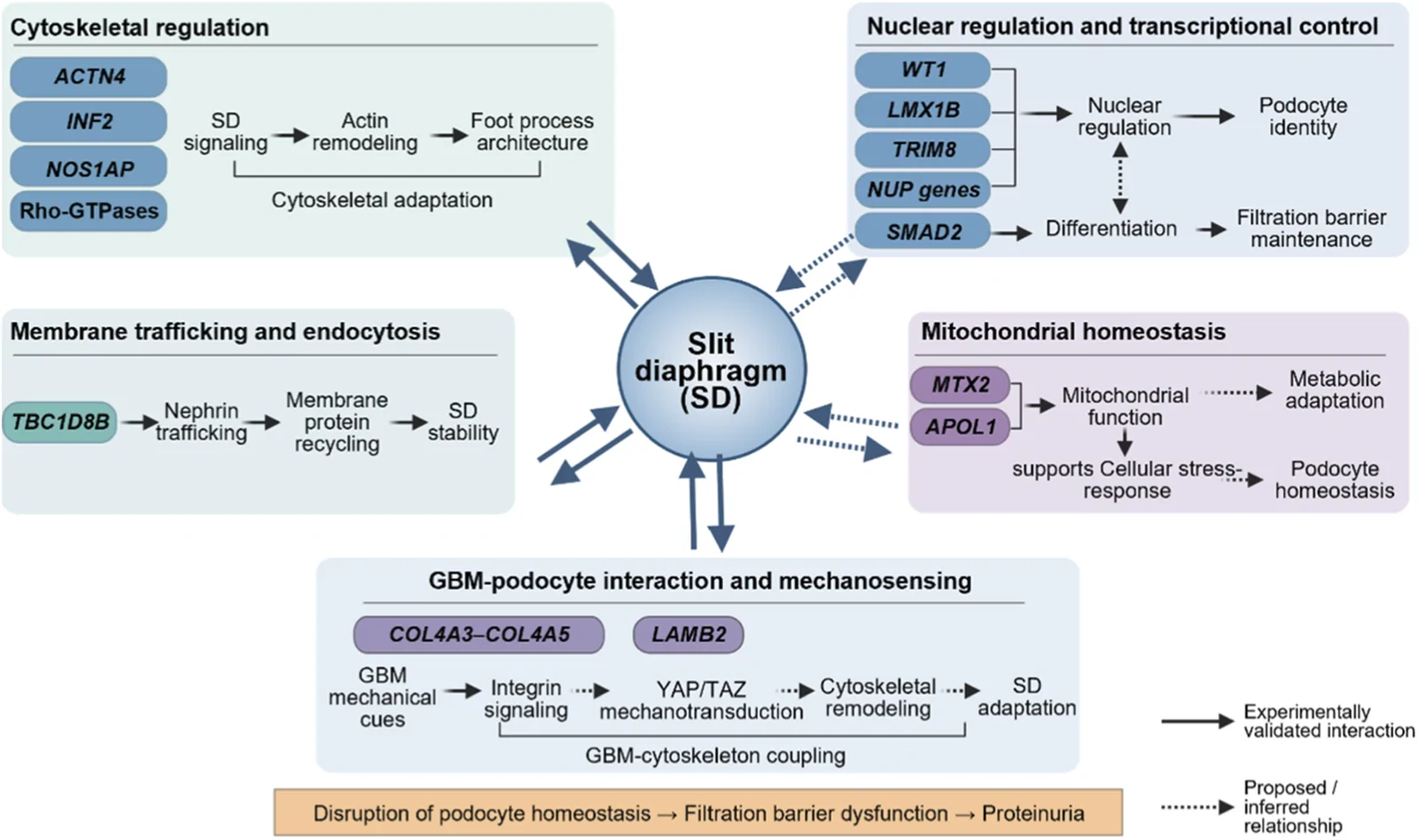
Integrated multi-layer network of genetic and cellular mechanisms underlying podocyte injury and proteinuria. Genetic factors associated with proteinuric kidney disease can be functionally organized into several interconnected regulatory layers, including cytoskeletal regulation (*ACTN4, INF2, NOS1AP, DAAM2, MYO9A, ARHGAP32*), membrane trafficking (*TBC1D8B*), nuclear and developmental regulation (*TRIM8, NUP* genes*, WT1, LMX1B, SMAD2*), mitochondrial homeostasis (*MTX2* and *APOL1*), and GBM–podocyte interaction (*COL4A3–COL4A5* and *LAMB2*). These pathways converge on shared cellular processes, including actin cytoskeleton remodeling, slit diaphragm stability, transcriptional regulation, metabolic adaptation, and mechanosensory responses. The integrated network illustrates how genetically diverse perturbations may disrupt coordinated podocyte homeostasis, ultimately leading to filtration barrier dysfunction and proteinuria.

Within such a system, disturbances need not remain confined to their point of origin. Alterations in one layer may propagate through successive levels, gradually affecting overall cellular function. For example, impaired trafficking of slit diaphragm proteins may modify cytoskeletal organization, while changes in nuclear regulation may alter expression of key structural or trafficking components (Chugh, 2007; Nina et al., 2009). Although direct causal links between all pathways have not been fully established, the available evidence supports functional interdependence.

This network perspective does not imply the existence of a single unifying molecular pathway. Instead, it suggests that different perturbations may converge at the level of cellular homeostasis. Such convergence may account for the recurring observation that diverse genetic variants produce overlapping phenotypes, including proteinuria and focal segmental glomerulosclerosis.

Several limitations of this network-based framework should be acknowledged. The current model remains largely qualitative, with node-specific weights, perturbation thresholds, and compensatory capacity of parallel pathways not yet defined. In addition, the framework is primarily podocyte-centered and does not systematically incorporate contributions from endothelial, mesangial, or circulating factors. Finally, the temporal dynamics of network disruption remain poorly understood, as most available data capture static states rather than the sequence of perturbation propagation across layers.

Beyond these model-intrinsic limitations, functional characterization of newly identified disease-associated variants often lags behind their clinical discovery, creating a gap between diagnostic sensitivity and mechanistic understanding. The podocyte-centered framework also remains incomplete because early podocyte injury can rapidly alter communication within the broader glomerular microenvironment (Clark et al., 2022).

Importantly, genetic variants should not be interpreted as isolated molecular abnormalities but rather as initiating events that perturb protein-level organization within the podocyte network. A disease-associated variant may alter protein conformation, stability, localization, or interaction capacity, which subsequently affects molecular assemblies and regulatory pathways (Roselli et al., 2010). For example, mutations affecting slit diaphragm proteins may impair their interaction networks or membrane organization, whereas variants in cytoskeletal regulators may alter actin dynamics and mechanical adaptation (Kaplan et al., 2000; Brown et al., 2010). Therefore, the pathogenic trajectory of podocytopathies can be viewed as a progressive transition from genetic perturbation to protein dysfunction, network instability, and cellular phenotype.

Although the proposed podocyte-centered network framework is centered on genetic mechanisms, it also provides a context for understanding how acquired insults may converge on molecular nodes that are vulnerable to genetic perturbation. Genetic variants represent one type of initiating perturbation, whereas acquired insults may affect shared vulnerable molecular nodes through distinct mechanisms. For example, genetic defects affecting slit diaphragm proteins and acquired immune-mediated disruption of nephrin function may both compromise filtration barrier integrity (Kestilä et al., 1998; Watts et al., 2022), while metabolic and mitochondrial stresses may converge with genetic mitochondrial defects to impair cellular adaptation (Alicic et al., 2017; Coughlan et al., 2016). Thus, non-genetic mechanisms provide complementary evidence that diverse perturbations may converge on shared vulnerabilities within the podocyte network. Pharmacological interventions are discussed selectively where they provide functional evidence that molecular pathways implicated by genetic studies are biologically relevant and potentially modifiable, rather than as an independent focus of the review.

### 7.4 Implications of a network-based framework

Interpreting podocyte biology through a network-based lens offers a way to reconcile several features of proteinuric disease that are difficult to explain within traditional models. Genetic heterogeneity, variable disease progression, and partial therapeutic responses may reflect differences in the specific nodes or connections affected within the network, rather than distinct disease categories.

This perspective also suggests that effective intervention may not require correction of a single molecular defect. Modulation of key regulatory nodes, stabilization of critical interactions, or restoration of balance across interconnected pathways could potentially influence disease progression. At present, such approaches remain largely conceptual, and further work is needed to define which elements of the network are most amenable to therapeutic targeting.

Although this framework is supported by recent genetic and experimental observations, its broader applicability requires validation in patient-derived systems and integrated multi-omics analyses. Clarifying how specific perturbations propagate across cellular layers will be essential for translating these concepts into clinical practice. If diverse genetic perturbations converge on shared network vulnerabilities, then identifying which functional node is affected in an individual patient becomes essential for mechanism-based stratification. This conceptual link connects the network model directly to the diagnostic and translational considerations discussed below.

## 8 Clinical implications and translational perspectives

### 8.1 Toward mechanism-oriented genetic diagnostics

Recent advances in gene discovery have begun to reshape diagnostic strategies for proteinuric kidney diseases. Conventional genetic testing has largely focused on structural components of the filtration barrier, including *NPHS1, NPHS2, WT1, COL4A3–COL4A5,* and *LAMB2*. While these panels capture a subset of monogenic cases, accumulating evidence indicates that a significant proportion of patients harbor variants in genes associated with intracellular trafficking, nuclear regulation, or mitochondrial function (Kaur et al., 2023; Cheng et al., 2024). Consistent with this, whole-exome sequencing in a multicenter pediatric nephrotic syndrome cohort identified genetic changes associated with distinct clinical phenotypes, supporting the diagnostic value of broad genetic testing beyond classical podocyte structural genes (Jiao et al., 2022).

High-throughput sequencing approaches, including whole-exome and whole-genome sequencing, have improved diagnostic yield, with pathogenic variants identified in approximately 25%–30% of patients with steroid-resistant nephrotic syndrome (Kaur et al., 2023; Cheng et al., 2024). More recent whole-genome sequencing studies suggest that higher diagnostic yields may be achievable in selected cohorts. For example, a 2025 study of 41 families with early-onset SRNS reported a monogenic diagnostic yield of 56.1% (Soliman et al., 2025), while a parallel investigation of a highly admixed pediatric population identified a combined prevalence of Mendelian variants and *APOL1* high-risk genotypes of approximately 20%, including novel variants in individuals of Native American ancestry (Watanabe et al., 2024).

These updated estimates suggest that monogenic causes may be more prevalent than previously appreciated in early-onset steroid-resistant nephrotic syndrome and support broader application of genome sequencing in this population. At the same time, the substantial proportion of cases that remain unexplained highlights the need to investigate noncoding variation as well as non-genetic contributors.

However, detection remains incomplete, particularly for variants located in noncoding regions or affecting transcript processing. Findings related to loci such as *NOS1AP-C1orf226* suggest that intronic and regulatory variants may contribute to disease mechanisms and may require complementary approaches, including RNA sequencing, for accurate interpretation (Buerger et al., 2025).

These observations support a shift from fixed gene panels toward adaptive diagnostic strategies that reflect evolving mechanistic understanding. Rather than grouping genes solely by structural location, classification based on functional pathways may provide a more informative framework for interpretation.

### 8.2 Mechanism-based stratification of proteinuric disease

The expanding range of implicated genes raises the possibility of stratifying proteinuric diseases according to underlying mechanisms. Although such classifications are not yet standardized, a functional grouping can be proposed based on current evidence.

Variants affecting membrane trafficking pathways, exemplified by *TBC1D8B*, are associated with altered nephrin turnover and membrane protein distribution (Kampf et al., 2019; Milosavljevic et al., 2022). Disruption of these processes may lead to progressive instability of the slit diaphragm. Notably, similar functional consequences may arise in non-genetic contexts, as illustrated by nephrin-targeting autoantibodies (Hengel et al., 2024).

Alterations in nuclear regulatory components, including nucleoporins such as *NUP133* and *NUP160* or transcriptional regulators such as *TRIM8*, suggest that some forms of podocyte dysfunction may originate from disturbances in gene expression and nuclear organization (Weng et al., 2021; Rogg et al., 2022; Li Y. et al., 2024). These mechanisms differ conceptually from those affecting structural components at the cell surface.

Mitochondrial-associated pathways, including those involving *MTX2*, highlight the contribution of metabolic and structural regulation within the cell (Li T. et al., 2024). Unlike classical CoQ10 deficiency, these mechanisms extend beyond energy production and may influence multiple aspects of cellular behavior.

These categories are not mutually exclusive, and individual patients may exhibit overlapping features. At present, such stratification should be regarded as a conceptual tool rather than a definitive classification system.

### 8.3 Translational directions and therapeutic considerations

The identification of new genetic mechanisms has implications for the development of targeted interventions. One emerging direction involves expanding genetic analysis beyond coding regions. As demonstrated by studies of intronic and splice-altering variants, clinically relevant alterations may reside outside conventional sequencing targets (Rogg et al., 2022). Incorporating transcriptomic data may therefore enhance diagnostic precision.

A second area of development lies in the integration of multi-omics data. Combining genomic, transcriptomic, spatial, and functional datasets may allow reconstruction of the sequence of events linking genetic perturbations to cellular dysfunction and clinical phenotype (Li T. et al., 2024; Abedini et al., 2024). Recent single-cell multi-omic and spatial profiling of human kidneys further demonstrates how disease progression can be interpreted through spatially organized cellular states and microenvironmental interactions (Abedini et al., 2024). Patient-derived organoid systems and *in vitro* models are likely to play a central role in this process.

Therapeutic strategies may also evolve in response to these insights. Interventions targeting membrane trafficking, mitochondrial function, or immune-mediated disruption of slit diaphragm proteins are currently under investigation (Hengel et al., 2024; Ding et al., 2023). In parallel, gene-based approaches, including adeno-associated virus–mediated delivery systems, have shown proof-of-concept efficacy in experimental models (Ding et al., 2023).

The network framework described above also raises several experimentally testable questions. For example, whether slit diaphragm condensate stability can serve as a functional readout for small-molecule screening may now be explored using high-throughput platforms capable of identifying condensate-modulating compounds (Mayfield et al., 2025). In addition, podocyte-specific Rho GTPase interactors, such as KIAA1522, may represent potential biomarker candidates, particularly in the context of urinary extracellular vesicles, which have been shown to correlate with disease activity in glomerular injury (Alves et al., 2025). Furthermore, YAP/TAZ target gene signatures in podocytes may provide a functional readout of mechanical stress experienced at the glomerular filtration barrier (Rinschen et al., 2017; Chen et al., 2022). Whether these network nodes can be safely and effectively modulated remains to be determined.

Systematic evaluation of these and other network-derived hypotheses will be required to determine whether the proposed multi-layer framework can inform not only mechanistic interpretation but also clinical decision-making.

At present, most of these approaches remain at an early stage. Further work is required to determine which components of the regulatory network can be modified safely and effectively in human disease.

## 9 Conclusion

Recent genetic studies have substantially broadened the framework for understanding glomerular proteinuria. Rather than arising solely from defects in discrete structural elements, proteinuria is increasingly recognized as the outcome of disturbed coordination across interconnected cellular systems.

Evidence accumulated over the past 5 years indicates that podocyte function depends on coordinated regulation spanning multiple layers, including cell–matrix interaction, cytoskeletal organization, membrane trafficking, nuclear control, and mitochondrial activity. These processes do not operate in isolation. Alterations affecting one component may influence others, leading to progressive disruption of filtration barrier function.

Within this context, the concept of a multi-layer regulatory network provides a useful way to interpret both genetic heterogeneity and phenotypic convergence. Diverse mutations, despite affecting distinct molecular pathways, may produce similar clinical manifestations by perturbing shared aspects of cellular homeostasis.

Although glomerular proteinuria involves contributions from multiple cell types, podocyte-associated mechanisms appear to represent a central axis through which many of these disturbances converge. Understanding how different forms of genetic variation influence this regulatory network remains a key challenge.

Future progress will depend on integrating genetic findings with functional and systems-level analyses. By placing genetically diverse perturbations within a common multi-layer network framework, future studies may clarify how distinct molecular defects converge on shared pathological outcomes in podocytopathies. Understanding how perturbations propagate across cellular layers may provide a basis for mechanism-oriented classification and, ultimately, targeted therapeutic strategies.
